# Nest trees of northern spotted owls (*Strix occidentalis caurina*) in Washington and Oregon, USA

**DOI:** 10.1371/journal.pone.0197887

**Published:** 2018-05-31

**Authors:** Randall J. Wilk, Damon B. Lesmeister, Eric D. Forsman

**Affiliations:** 1 Department of Agriculture, Forest Service, Forestry Sciences Laboratory, Pacific Northwest Research Station, Olympia, Washington, United States of America; 2 Department of Agriculture, Forest Service, Forestry Sciences Laboratory, Corvallis, Oregon, United States of America; 3 Department of Fisheries and Wildlife, Oregon State University, Corvallis, Oregon, United States of America; National Cheng Kung University, TAIWAN

## Abstract

The northern spotted owl (*Strix occidentalis caurina*) is a federally-threatened subspecies in the United States associated with late-successional forests. In mesic forests it nests primarily in tree cavities, but also uses various types of external platform nests in drier forests. We describe 1717 northern spotted owl nests in 16 different tree species in five study areas in Washington and Oregon in the Pacific Northwest, USA. The vast majority of nests (87%) were in Douglas-fir (*Pseudotsuga menziesii*) trees, except on the Olympic Peninsula, Washington, where nests were about equally abundant in Douglas-fir, western red cedar (*Thuja plicata*), and western hemlock (*Tsuga heterophylla*) trees. Distribution of nests was 57.9% in top cavities of trees with broken tops, 20.3% in side cavities of hollow tree trunks, and 21.8% on external platforms of trees. Platforms were most common in the two driest study areas in the Eastern Cascades Physiographic Province, Washington (89% of nests), and the Klamath Province, Oregon (32%). The vast majority (89%) of nests were in trees with intact or declining crowns. Nests in dead trees were most common on the Olympic Peninsula. Nest trees with top and side cavities were larger and much more prevalent in study areas where annual precipitation was highest (Olympic Peninsula, Oregon Coast Range). Large nest cavities and platforms used by northern spotted owls occur almost exclusively in old forest. Managing for the retention of such forests and for their replacement is a significant challenge for land managers, especially in the face of climate change and an increasing human population, but will likely be required for the persistence of viable populations of northern spotted owls.

## Introduction

The northern spotted owl (*Strix occidentalis caurina*) was federally listed as a threatened subspecies in 1990 in the United States, primarily because of declining habitat and evidence of declining population trends [1]. Since that time, the subspecies has continued to decline, partly due to continued habitat loss, but also because of increasing competition with the invasive barred owl (*Strix varia*) [2, 3]. Northern spotted owls (hereafter, NSOs) are specialist predators on arboreal and scansorial forest mammals [4–6] and are most abundant in mature and old-growth forests throughout their range [7]. Because of the economic and ecological implications surrounding management of the NSO, it is one of the most studied birds in the world. Studies of associations between NSOs and forest cover consistently indicate a strong association with older forest conditions for nesting and roosting, with a wider range of forest cover types used for foraging and dispersal (e.g., [8–14]).

Nests of NSOs have been described in many areas of their range, including Oregon [8, 15], Washington [15–18], and northern California [19, 20]. Nesting by NSOs primarily occurs in hollow cavities or in external platforms in conifer trees infected by dwarf mistletoe (*Arceuthobium* spp.). Nesting on cliffs has been documented, but is rare [8, 21]. Nest site selection appears to depend primarily on availability of large, old trees and protective cover from predators and cold, wet weather during the early nesting season [8, 17, 18].

Most previous studies of NSO nesting were somewhat limited in spatial scope (i.e., single study area) or occurred before barred owls had fully colonized the entire geographic range of NSOs. Barred owls now outnumber NSOs in most areas and competitive interactions between the two species appear to represent a significant threat to the long-term persistence of the NSO [2, 3, 22, 23]. In Washington and Oregon, most documented nests come from sites on federal lands as a result of population monitoring studies (see [2, 24, 25]). These studies have provided a substantial understanding of NSO ecological requirements, and have informed forest management and species recovery plans [21, 26]. Our objectives were to characterize and compare NSO nest trees and nest types among five of the long-term demographic study areas in Washington and western Oregon, and show how precipitation may influence the types of nests used across the wide latitudinal scale. Likely driven primarily by availability within each region, we expected Douglas-fir (*Pseudotsuga menziesii*) and tree cavities to be the most commonly used tree species and nest type in areas with higher amounts of precipitation, and platform nests and other tree species collectively more frequent in drier portions of the NSO range.

## Materials and methods

### Statement

Data reported were in conjunction with the long term mark-recapture study of NSO demography [2]. All handling and tagging of northern spotted owls was authorized under the U.S. Fish and Wildlife Service Endangered Species 10a1a permit (Permit #TE-026280-15) and in compliance with the Oregon State University Animal Care and Use Permit (#4132). Scientific Collection Permits were obtained annually from Oregon Department of Fish and Wildlife and Washington Department of Fish and Wildlife, and a Federal Bird Banding Permit (#21249) was obtained from the U.S. Geological Survey, Bird Banding Lab. Permissions to access field sites were provided by United States (U.S.) Forest Service, U.S. Bureau of Land Management, U.S. Park Service, Oregon and Washington land management agencies, and many private landowners.

### Study area

**Fig 1:**
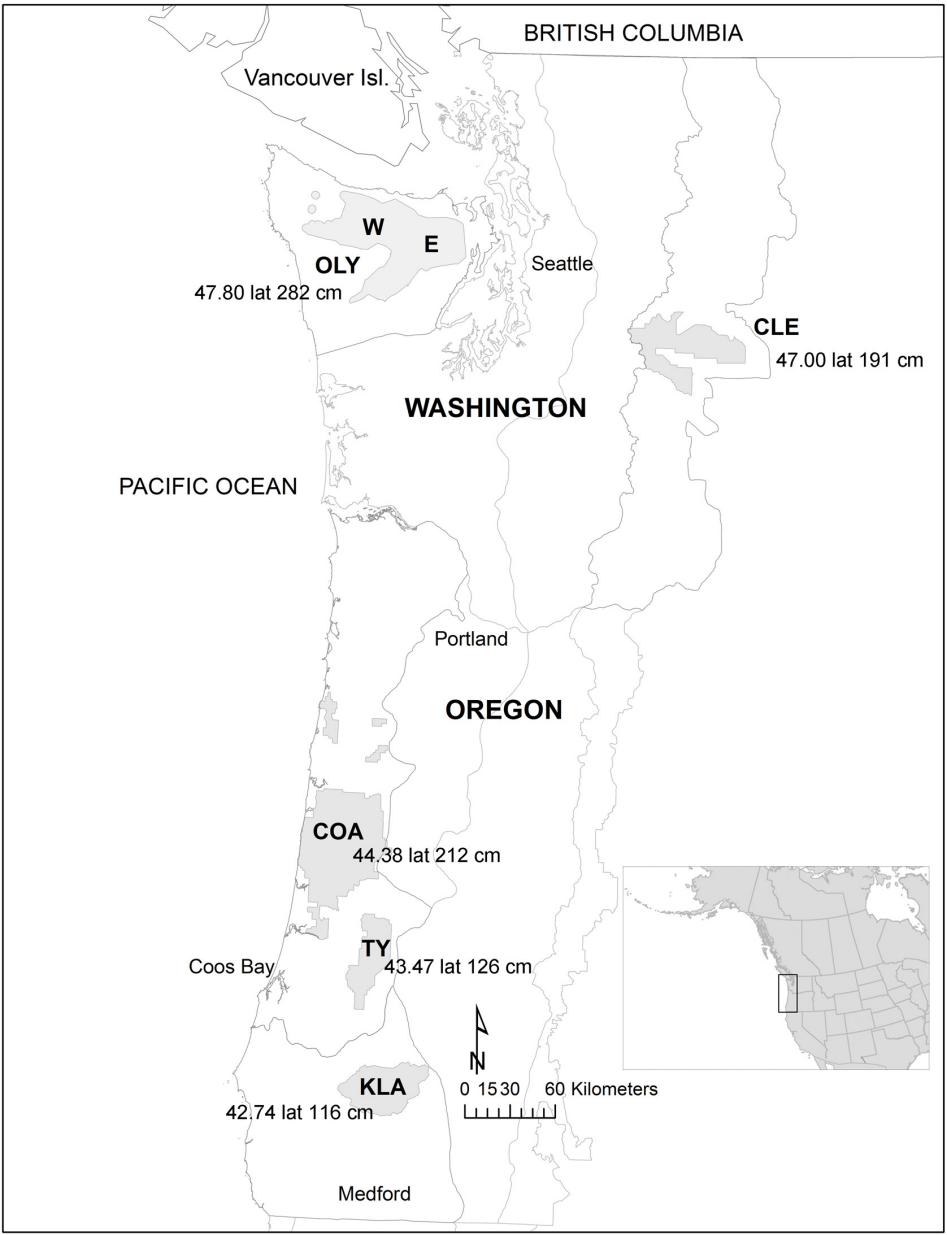
Locations in Washington and Oregon, United States of study areas with nest trees of northern spotted owls, 1985 to 2013. Study areas (Washington: OLY, CLE; Oregon: COA, TYE, KLA) are shaded and gray lines show borders of the larger physiographic provinces. Shown with study area identifiers are latitude (lat) and mean annual precipitation (cm). The longitudinal range of the study areas spanned 120°–124° W. OLY was subdivided between the western side (W) and eastern side (E).

We used NSO nesting data collected from 1985–2013 in five study areas of four physiographic provinces (ecologically similar areas) in Washington and Oregon where demography monitoring occurred (Fig 1). Details on how nests were located are in [2, 27–29]. Three study areas, Coast Ranges (COA) and Tyee (TYE) of the Coast Range Physiographic Province, and Klamath (KLA) of the Klamath Province (approximately 42° 74’ N to 44° 38’N, and 123°–124° W), Oregon were located in regions dominated by subclimax forests of Douglas-fir [30]. One study area, Cle Elum (CLE), of the Eastern Cascades Province (approximately 47° 00’ N, 120° W), was located on the east slope of the Cascades Range, Washington, where mixed species forests of Douglas-fir, ponderosa pine (*Pinus ponderosa*) and grand fir (*Abies grandis*) trees predominated [31]. The fifth study area was on the Olympic Peninsula (OLY) of the Olympic Peninsula Province, Washington (approximately 47° 80’ N, 124° W), subdivided into western (OLY W) and eastern (OLY E) subprovinces [17] (Fig 1), with climax rain forests of western hemlock (*Tsuga heterophylla*), western red cedar (*Thuja plicata*), and coastal Sitka spruce (*Picea sitchensis*) trees, which dominated much of the western half of the peninsula. Annual precipitation varied by study area (Fig 1) and occurred mostly as rain with snow at high elevations [30].

### Data analyses

**Fig 2:**
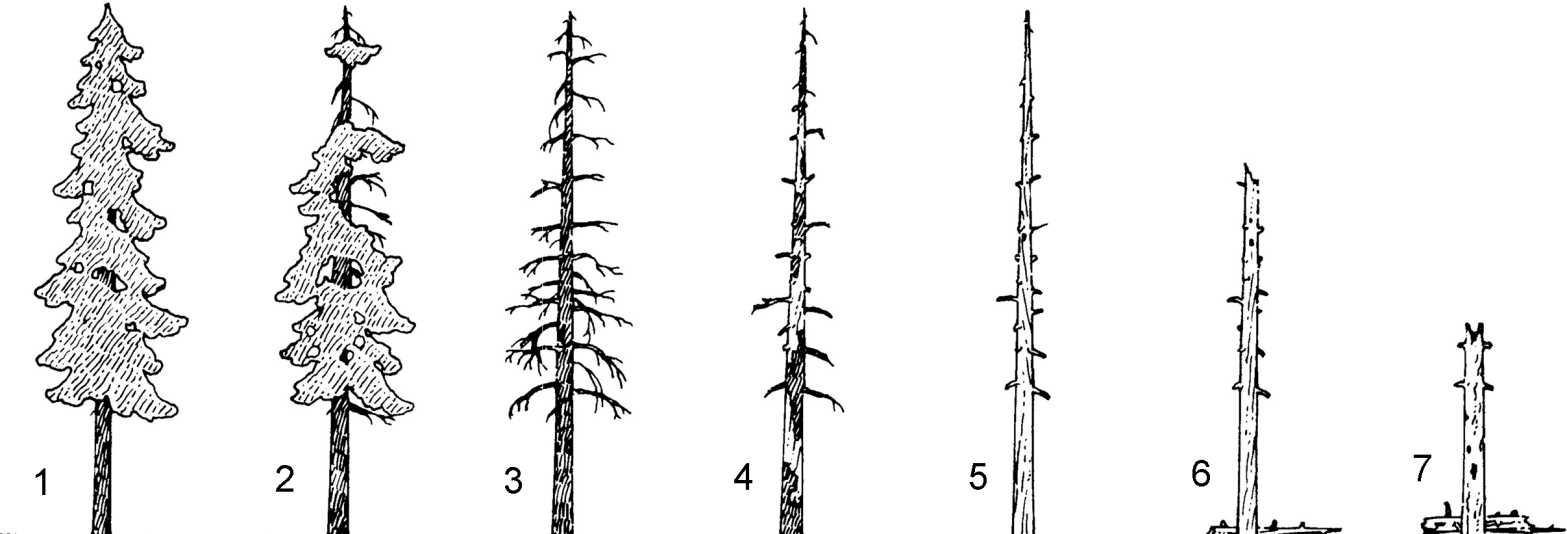
Decomposition stage time sequence continuum of nest trees of northern spotted owls, northwestern United States. Stage number, description, and approximate range of years since tree death are as follows: stage 1–intact crown and loosened bark, 0–6 years; stages 2 and 3–declining crown, broken tops, soft wood, 7–18 years; stage 4–loose bark and wood, only stubs of large limbs remain, 19–50 years; stages 5 and 6–no bark, accumulated wood and bark around tree base, 51–125 years; stage 7–decomposed, ≥ 125 years [32, 33]. Stages 1–2 are considered alive, and stages 3–7 are dead trees. Images are not scaled.

We summarized and compared nest tree variables across study areas, which included proportional compositions of species, stage of succession or decomposition [32, 33] (Fig 2), nest type, diameter at breast height (DBH), total height, and height to the nest (COA only). We computed nest tree alpha diversity for each study area with the Shannon-Weiner Index (*H*) where higher values indicate that numbers of individual nest trees in the total of nest trees are more equitably distributed among the nest tree species. Nest types were divided into three categories that included nests that were accessed via the broken tops of hollow trees (top cavities), nests inside hollow trees that were accessed by holes in the side of the trunk (side cavities), and nests in external platforms that were constructed by other wildlife, or that formed naturally when debris collected on limbs or with dwarf mistletoe infections (platforms).

To compare variables across study areas, we used 95% confidence intervals (CIs) and error bar overlap inference rules of eye to estimate statistical significance [34, 35]. Error bars provide more information than test statistics and *P*-values, including effect size and precision of estimates, or the uncertainty attendant to interpretation of results [35]. Error bar overlap to about one-half plus the length of an error bar (0.59 overlap) is *P* ≈ 0.05, and for the area between slight overlap of error bars to one-half arm length is *P* ≤ 0.05. If the error bars are slightly touching (≤ 0.14 overlap), *P* = 0.01. If the gap between bars is approximately one-third the length of a single error bar (–0.37 gap), *P* ≈ 0.001, and if greater than one-third the length of a single error bar, *P* < 0.001 [34]. Error bars are asymmetrical when *P* is closer to 0 or 1.

We created a combined space ordination matrix to display dissimilarity between study areas by ranked distance based on percentages composition of nest types and of the three main species of nest trees using nonparametric multidimensional scaling with PC-ORD, v.5.31 [36]. We ordinated the compositions on study area and environmental variables, latitude and average annual precipitation [2] (Fig 1). For OLY subprovinces we averaged the long term average annual rainfall from weather reporting locations from the Western Regional Climate Center [37]. Prior to analysis, we applied the arcsine square root transformation to more normalize proportion data [36]. We used PC-ORD default settings and conducted multiple runs to evaluate model fit [38]. We orthogonally rotated the ordination to load the environmental variable with the highest correlation value on the horizontal axis to help improve interpretation [36]. Resulting correlation coefficients are approximate indicators of significance. We used 95% CIs for Pearson’s *r* correlations for variables with the ordination axes to assess the strength of relationships, where an *r* > │0.9│is a very strong association or effect, *r* = │0.7 to 0.9│ is strong, *r* = │0.5 to 0.7│is moderately strong, *r* = │0.3 to 0.5│is low strength, and *r* = │< 0.3│is little if any correlational association [39]. If the CI lower limit (CI LL) was about ≥ 0.3 we considered the correlation to be biologically meaningful, and if about ≤ 0.3 we did not consider the association to be biologically meaningful. We used IBM SPSS Statistics (v. 24, 2016 IBM Corp.) to process data and compute tree size metrics with CIs, and followed [40] to compute CIs for proportions (percentages) and correlation coefficients.

## Results

### Nest types

**Fig 3:**
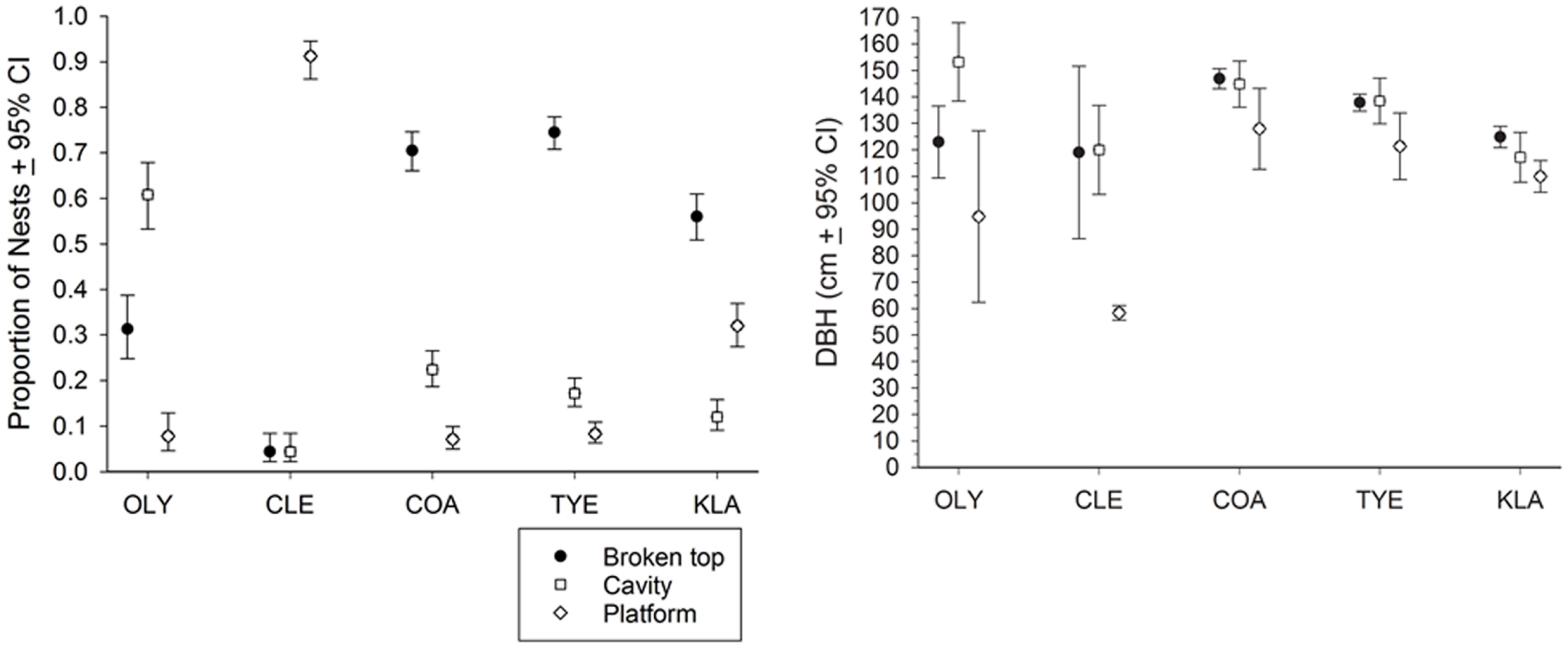
Proportion (L) and diameter at breast height (R) of nest trees of northern spotted owls in Washington and Oregon, United States, subdivided by study area and nest type.

Of 1717 nest trees located during the study, 57.9%, CI = 56–60%, had nests in top cavities, 20.3%, CI = 18–22%, had nests in side cavities, and 21.8%, CI = 20–24%, had nests in external platforms. The distribution of nest types was very different among study areas, with top cavities or side cavities predominating in the OLY, COA, and TYE study areas, and platform nests were much more common in the drier CLE and KLA study areas (Fig 3). Side-cavity nests were overall most common on OLY. Compared to the other study areas OLY E had unique similar proportions of the three nest types in decomposition stages 1–2 (range 15.3–23.7%), but had a significantly larger proportion of broken-top nests in dead trees (stages 3–7) than the other study areas (25.4%, CI = 16–38%; range of other study areas = 2.2–10.8%; *P* = 0.01 to << 0.001) (S1 Table).

There were large differences in the types of nests on the OLY subprovinces. OLY E had greater proportions of top cavity (49.2%, CI = 37–62%) and platform (15.3%, CI = 8–27%) nests than OLY W (21.5%, 15–30%, *P* << 0.001 and 3.7%, 2–9%, *P* < 0.05, respectively) but in OLY W (74.8%, CI = 66–82%), side-cavity nests were > OLY E (35.6%, CI = 25–48%; *P* << 0.001) (S1 Table). In OLY E, top-cavity (*P* << 0.001) and side-cavity (*P* < 0.05) nests were > platform nests. In OLY W, side- and top-cavity nests were > platform nests (both *P* << 0.001) (S1 Table).

### Tree species

**Fig 4:**
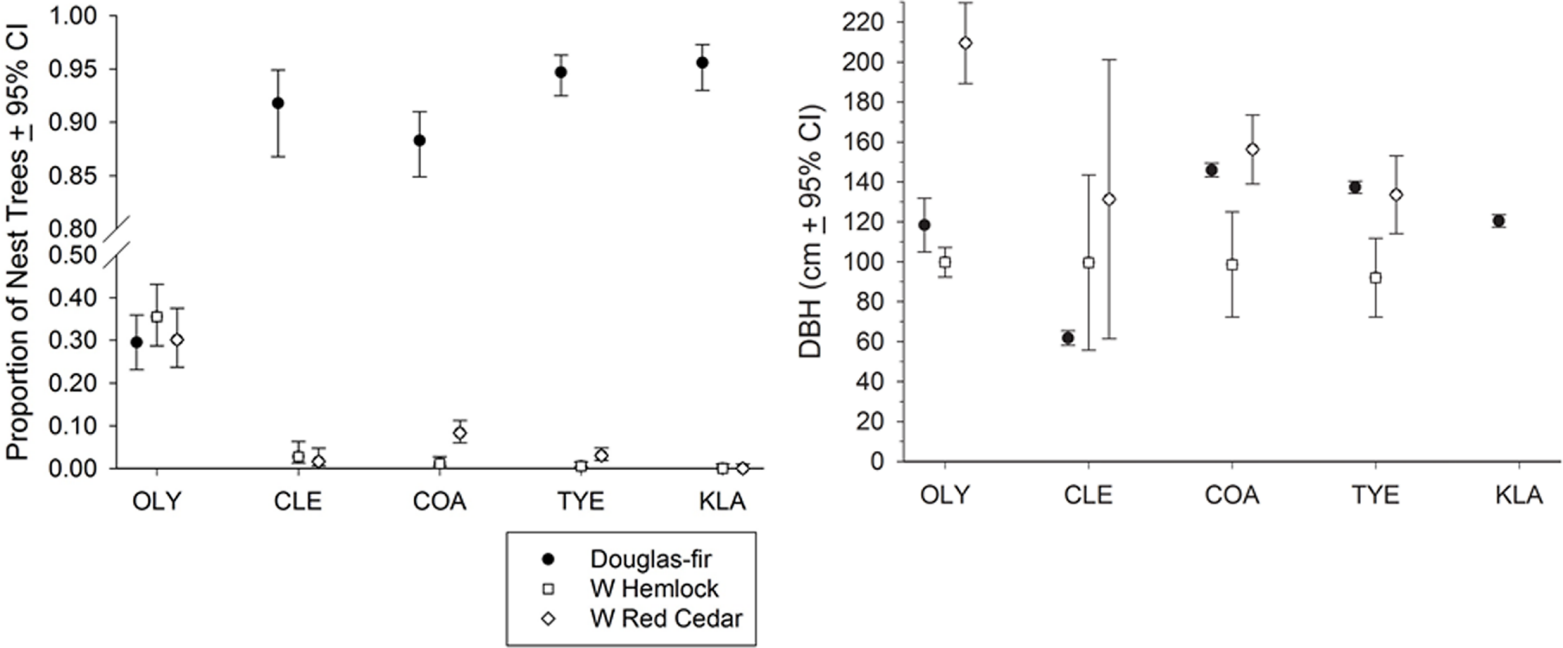
Proportion (L) and diameter at breast height (R) of nest trees of northern spotted owls in Washington and Oregon, United States, subdivided by study area and three important species. The three tree species comprised 97% of the nest trees.

Douglas-fir (87%, CI = 85–88%), accounted for the vast majority of nest trees on all study areas except for OLY, where nests were about equally abundant in Douglas-fir (CI = 23–37%), western red cedar (CI = 24–38%) and western hemlock (CI = 29–43%) trees. Forty-seven percent of the western red cedar nest trees and 82% of the western hemlock nest trees were on OLY. In KLA there were no nests in either of the two species. The evenness of nest tree composition on OLY, especially OLY E, resulted in the highest nest tree diversity (1.307), and among study areas, diversity increased with increasing latitude (Tables 1 and 2, Fig 4). Only the three western Oregon study areas had nests in hardwood trees and hardwood nest tree species *H* decreased with increasing latitude: Hardwood nest tree species *H* only in KLA = 0.693, in TYE = 0.562, and in COA = 0.451.

**Table 1:** Species and number of nest trees of northern spotted owls in Washington and Oregon, USA, subdivided by study area or subprovince.

		OLY E	OLY W	CLE	COA	TYE	KLA	Total
Douglas-fir	*Pseudotsuga menziesii*	34	15	167	385	535	350	1486
Western hemlock	*Tsuga heterophylla*	14	45	5	5	3	0	72
Western red cedar	*Thuja plicata*	6	44	3	36	17	0	106
Incense-cedar	*Calocedrus decurrens*	0	0	0	1	5	6	12
Grand fir	*Abies grandis*	1	1	4	1	1	1	9
Bigleaf maple*	*Acer macrophyllum*	0	0	0	5	1	0	6
Pacific madrone*	*Arbutus menziesii*	0	0	0	1	3	2	6
Ponderosa pine	*Pinus ponderosa*	0	0	2	0	0	2	4
Sitka spruce	*Picea sitchensis*	0	1	0	2	0	0	3
Pacific silver fir	*Abies amabilis*	2	1	0	0	0	0	3
Sugar pine	*Pinus lambertinana*	0	0	0	0	0	2	2
Gary white oak*	*Quercus kelloggii*	0	0	0	0	0	2	2
Mountain hemlock	*Tsuga mertensiana*	1	0	0	0	0	0	1
Western larch	*Larix occidentalis*	0	0	1	0	0	0	1
Jeffrey pine	*Pinus jeffreyi*	0	0	0	0	0	1	1
Western white pine	*Pinus monticola*	1	0	0	0	0	0	1
Totals	* *	59	107	182	438	565	366	1717
*H* diversity	* *	1.213	0.610	0.561	0.485	0.277	0.256	

*Hardwood species.

**Table 2:** Percentages of nest trees of northern spotted owl in Washington and Oregon, USA, subdivided by species, alive/dead decomposition stages and nest type. Parentheses show 95% confidence intervals (*n* = 1717).

Species	Decomposition stages 1–2 alive trees			Decomposition stages 3–7 dead trees		
	Platform	Side cavity	Top cavity	Platform	Side cavity	Top cavity
Douglas-fir among all nest trees	19.6 (18–22)	9.0 (8–11)	49.5 (47–52)	0.3 (0.1–0.7	2.9 (2–4)	5.2 (4–6)
Within Douglas-fir nest trees	22.7 (21–25)	10.4 (9–12)	57.2 (55–60)	0.3 (0.1–0.8)	3.3 (3–4)	6.1 (5–7)
Western red cedar among all nest trees	0.1 (0–0.4)	3.3 (3–4)	1.3 (0.8–1.9)	0.1 (0–0.3)	1.2 (0.8–1.8)	0.2 (0.1–0.6)
Within western red cedar nest trees	1.9 (0.5–7)	53.8 (44–63)	20.8 (14–29)	0.9 (0.2–5)	18.9 (13–27)	3.8 (2–9)
Western hemlock among all nest trees	0.3 (0.1–0.7)	2.2 (2–3)	0.6 (0.4–1.1)	0 (0–0.2)	0.8 (0.4–1.3)	0.3 (0.1–0.7)
Within western hemlock nest trees	6.9 (3–15)	52.8 (41–64)	15.3 (9–25)	0 (0–5)	18.1 (11–29)	6.9 (3–15)
All other species among all nest trees	1.4 (0.9–2.1)	0.9 (0.5–1.4)	0.5 (0.3–1)	0 (0–0.2)	0.1 (0–0.3)	0.2 (0.1–0.6)
Within all other 13 species of nest trees	45.3 (33–59)	28.3 (18–42)	17.0 (9–29)	0 (0–7)	1.9 (0.3–10)	7.5 (3–18)
Total	21.4 (2–23)	15.4 (14–17)	52.0 (50–54)	0.3 (0.2–0.8)	4.8 (4–6)	6.0 (5–7)

### Tree decomposition stage

The vast majority (88.8%, CI = 87–90%) of nest trees had declining (stage 2) or intact (stage 1) crowns, and the rest (11.2%, CI = 10–13%) were in advanced decomposition stages 3–7 (dead) (S1 Table). Most of the live nest trees (stages 1 or 2) had declining crowns (stage 2), especially in cases of trees with top-cavity or side-cavity nests. Platform nests were more likely to be in trees with stage 1 intact, healthy crowns (CI = 80–90%; *P* << 0.001; Table 3).

**Table 3:** Percentages of decomposition stages of nest trees of northern spotted owls in Washington and Oregon, USA, subdivided by study area, species, and nest types.

Stage description	Intact crown	Declining crown		Loose bark	No bark		Decom- posed
Stage number	1	2	3	4	5	6	7
Sample size (*n*)	295	1059	39	36	20	58	28
Study area							
OLY	13.9	62	7.2	9	6.6	1.2	0
OLY W	13.1	70.1	4.7	8.4	1.9	1.9	0
OLY E	15.3	22.3	11.9	10.2	15.3	0	0
CLE		94.0^a^					6.0^a^
COA	9.1	77.6	1.6	1.1	1.6	6.2	2.7
TYE	10.4	79.5	1.2	1.4	0.4	4.4	2.7
KLA	47.3	45.6	3.6	2.2	0	1.1	0.3
Tree species							
Douglas-fir	18.8	71	2.1	1.8	0.8	3.4	2
Western red cedar	7.8	68	1.9	4.9	5.8	10.7	1
Western hemlock	16.4	58.2	10.4	8.9	6	0	0
Other species	60.9	28.3	4.3	2.2	0	4.3	0
Nest type							
Broken top	5.3	84.7	2	1.6	1	3.7	1.6
Side cavity	19.1	57.1	5.3	5.9	2.9	6.2	3.5
Platform	85.6	13.9	0.5	0	0	0	0

^a^Values are stages 1–2 combined and stages 3–7 combined for CLE where only the pooled data were available.

Nest types in Douglas-fir trees followed a similar pattern as all trees combined, but in western red cedar and western hemlock trees, side-cavity nests were more common, followed by top-cavity and platform nests (Table 1). Among all other 13 species of nest trees, most of those nests were platforms in trees with intact crowns, followed by side-cavity and top-cavity nests (Tables 1 and 3).

### Tree size

Overall nest trees averaged 127.6 cm DBH, CI = 125–130 cm. From these, hardwood nest trees averaged 85.6 cm, 74–97 cm (*n* = 14). Total heights averaged 38.6 m, CI = 38–39 m, and hardwood nest trees averaged 25.9 m, CI = 23–29 m. Data on the height of nests was available only for COA, $\bar{x}$ = 27.0 m, CI = 26–28 m (*n* = 434). Six of these were in hardwoods, $\bar{x}$ = 11.8 m, CI = 5.1–18.5 m.

The largest nest trees (DBH and total height) were in COA, followed by OLY and TYE where side-cavity nests were most prevalent, and were larger than CLE and KLA (*P* << 0.001), where platform nests were most prevalent (Figs 3 and 4, Table 4; data vary from Table 5 due to different sample sizes). Tree size in COA was greater than TYE (*P* < 0.01). OLY W nest trees (DBH $\bar{x}$ = 156.1 cm, CI = 142–170 cm) were larger diameter than OLY E nest trees (DBH $\bar{x}$ = 108.5, CI = 96–121 cm, *P* << 0.001).

**Table 4:** Diameter at breast height and total height of nest trees of northern spotted owls by species in Oregon and Washington, USA, subdivided by study area. Diameters ($\bar{x}$ cm) and heights ($\bar{x}$ m) include 95% confidence intervals (parentheses). Due to small sample size, some CIs are not shown.

Species	Douglas-fir			Western red cedar			Western hemlock		
Measure	*N*	Diameter	Height	*N*	Diameter	Height	*N*	Diameter	Height
OLY									
Top cavity	26	127.0 (110–144)	36.0 (29–43)	9	172.3 (129–216)	45.0 (36–54)	14	86.6 (72–101)	33.9 (26–41)
Side cavity	16	114.9 (89–141)	36.4 (27–45)	40	218.2 (195–231)	47.6 (44–51)	42	106.6 (98–115)	39.7 (36–43)
Platform	7	94.4 (41–148)	37.1 (23–51)	1	198	53	3	65.3 (11–120)	38.0 (22–54)
CLE									
Top cavity	6	122.5 (83–162)	24.0 (9–39)	1	150	33	0		
Side cavity	3	117.0 (60–174)	31.7 (23–51)	2	122.0	28.5	3	121.7 (76–167)	30.0 (23–37)
Platform	158	58.4 (56–61)	32.3 (31–33)	0			2	66.5	35.0
COA									
Top cavity	296	147.2 (143–151)	41.6 (40–43)	8	159.8 (123–197)	34.5 (23–46)	1	95	36
Side cavity	64	145.5 (136–155)	39.7 (36–43)	26	157.6 (136–180)	36.9 (33–41)	4	99.5 (61–138)	42.3 (28–57)
Platform	25	132.6 (115–150)	47.2 (42–52)	2	125.5	49.0	0		
TYE									
Top cavity	412	137.9 (135–141)	38.5 (37–40)	8	138.4 (98–178)	33.3 (23–43)	1	95	36
Side cavity	79	145.5 (136–155)	39.7 (36–43)	9	129.4 (108–151)	35.6 (26–45)	2	90.5	45.0
Platform	44	124.1 (111–137)	46.3 (41–51)	0			0		
KLA									
Top cavity	200	125.6 (122–129)	36.0 (35–37)	0			0		
Side cavity	42	120.0 (111–129)	32.7 (29–37)	0			0		
Platform	108	111.3 (105–117)	42.9 (41–45)	0			0		
Total									
Top cavity	940	137.8 (136–140)	38.8 (38–40)	26	157.2 (137–177)	37.7 (32–43)	16	87.6 (75–100)	33.9 (27–40)
Side cavity	204	136.0 (131–141)	38.0 (36–40)	77	184.8 (169–202)	42.1 (39–45)	51	106.3 (99–114)	39.5 (36–43)
Platform	342	89.7 (85–94)	38.6 (37–40)	3	149.7	50.3	5	65.8 (41–91)	36.8 (27–46)

**Table 5:** Diameter at breast height and total height of nest trees of northern spotted owl in Oregon and Washington, USA, subdivided by decomposition stage. Diameters ($\bar{x}$ cm) and heights ($\bar{x}$ m) include 95% confidence intervals (parentheses). Data for CLE are missing.

Stage description	Intact crown	Declining crown		Loose bark	No bark		Decomposed	Total
Stage number	1	2	3	4	5	6	7	
Sample size	295	1059	39	36	20	58	28	1535
Height	42.8 (41–44)	41.4 (41–42)	27.5 (24–31)	21.5 (18–25)	26.6 (20–33)	20.7 (19–22)	15.7 (13–18)	39.4 (39–40)
range	11–79	6–76	12–53	7–54	7–50	7–41	8–35	6–79
Diameter	120.2 (116–124)	141.8 (139–144)	123.1 (110–136)	106.6 (97–116)	114.8 (93–136)	125.2 (115–136)	128.7 (87–217)	135.1 (133–137)
range	13–252	15–379	32–228	60–182	41–222	59–273	87–217	13–379

Nest trees in decomposition stage 2 had the largest diameters (*P* < 0.05), were of similar total height as stage 1 trees, and were taller than nest trees in decomposition stages 3–7 (*P* << 0.001). Trees in stages 3–7 were of similar size (Table 5).

The diameter of trees with top-cavity nests (DBH $\bar{x}$ = 137.1 cm, CI = 135–139 cm, *n* = 995) was similar to the size of trees with side-cavity nests (DBH $\bar{x}$ = 141.4 cm, CI = 136–147 cm, *n* = 348), and both were larger than trees with platform nests (DBH $\bar{x}$ = 89.4 cm, CI = 85–94 cm, *n* = 374; *P* << 0.001). Total height of trees among nest types was not different (top $\bar{x}$ = 38.5 m, CI = 38–39 m; side $\bar{x}$ = 38.9 m, CI = 38–40 m; platform $\bar{x}$ = 38.5 m, CI = 38–40 m).

Side-cavity nest trees on OLY were larger than the other nest types, and platform nests in CLE were smaller than the other nest types (Fig 3). In Oregon study areas (COA, TYE, KLA), the pattern of relative tree size across nest types was similar and trees with top cavities were larger than trees with platforms.

Overall, the largest nest trees were of western red cedar, ($\bar{x}$ = 177.1 cm DBH, CI = 164–190 cm), followed by Douglas-fir ($\bar{x}$ = 126.5 cm DBH, CI = 124–129 cm), and western hemlock ($\bar{x}$ = 99.4 cm, CI = 93–106 cm) trees.

The largest Douglas-fir nest trees were in COA. The largest western red cedar nest trees were on OLY, and western hemlock trees were not different in size among study areas (Fig 4). The tallest nest tree species were red cedar trees ($\bar{x}$ = 41.3 m, 39–44 m), which were taller than Douglas-fir trees ($\bar{x}$ = 38.7 m, CI = 38–39 m; *P* = 0.01). Western hemlock trees averaged 38.1 m, CI = 35–41 m.

Western red cedar (*P* < 0.01) and western hemlock (*P* < 0.05) side-cavity nest trees were larger diameter than top-cavity nest trees, but Douglas-fir trees with side-cavity and top-cavity nests were not different in size (Table 4).

### Ordination

**Fig 5:**
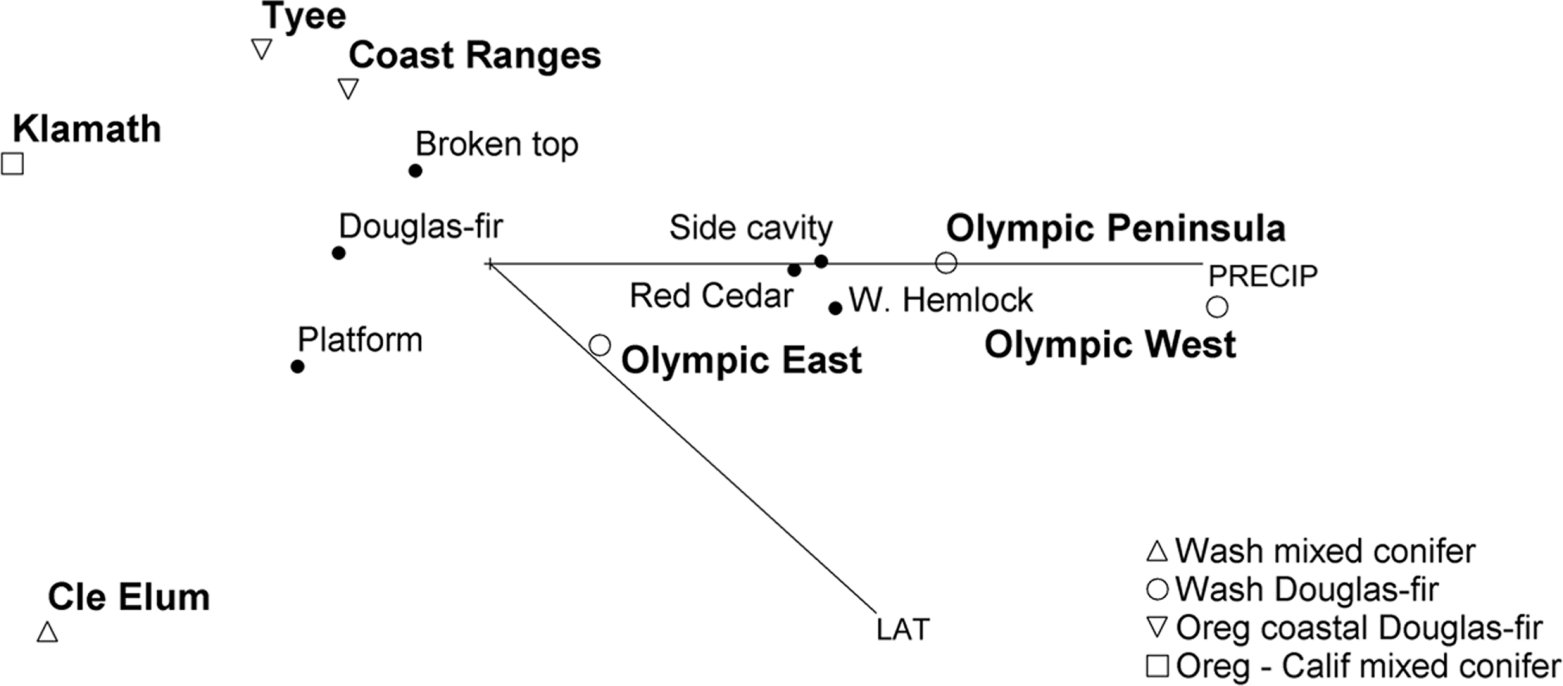
Nonparametric multidimensional scaling ordination of study areas in nest tree characteristics space of northern spotted owls, northwestern United States. The horizontal axis was rotated on precipitation (PRECIP). Lines show correlation vectors (radiating from the centroid, +) of environmental variables with the ordination (PRECIP and latitude [LAT]). Figs 3(L) and 4(L) depict proportions prior to transformation for ordination. The three tree species comprised 97% of the nest trees among 16 species.

The ordination demonstrates that tree species and nest type are not statistically independent and amount of precipitation has a strong influence on both (Fig 5). The horizontal axis accounted for 97.6% of the variation in the data while the vertical axis accounted for 15.8%. Precipitation (PRECIP) explained 88.7% of the variation along the horizontal axis and latitude (LAT) explained 48.1% of the variation. Side-cavity nests (*r* = 0.988, CI LL = 0.918†), western red cedar nest trees (*r* = 0.984, CI lower limit [LL] = 0.892***), Douglas-fir nest trees (*r* = –0.976, CI LL = –0.841***), western hemlock nest trees (*r* = 0.939, CI LL = 0.635**) reflect the relationship of tree species and nest type, precipitation (*r* = 0.942, CI LL = 0.650**), and association to OLY along the horizontal axis, and the LAT vector reflects the close association of top-cavity (broken top) nests (*r* = 0.896, CI LL = 0.439*) to COA and TYE in vertical space († = very strong association; *** = strong; ** = moderate; * = low strength). Distance between entities approximate the dissimilarity between them. The study areas are accurately represented in space because of their unique composition combinations of species and nest type, whereas tree species and nest type are an average or typical position ignoring the breadth of their distributions across the region.

## Discussion

Large trees with broken tops and hollow trunks are common in remnant areas of old conifer forests in western Washington and Oregon [41, 42]. These types of trees provided the vast majority of nests used by NSOs in our study and in most previous studies of NSOs. Large dead trees are also common in old conifer forests [33] and provided an important source of nest sites, especially on the Olympic Peninsula. Nests in external platforms were relatively unimportant in study areas characterized by high precipitation, but were the primary nest type on the east slope of the Cascades in Washington.

Side-cavity nests were especially prevalent in western red cedar and western hemlock trees in OLY W where the highest precipitation occurred, and were also common in the mesic forests of the Oregon Coast Range Province. NSOs may select side-cavity nests because they provide more protection from the frequent heavy precipitation that occurs during the nesting season [8, 17]. Our findings support the side-cavity nest selection hypothesis for higher level of protection in areas with cold and rainy conditions during the breeding season, but we cannot discount the possibility that our results may be a reflection of higher availability of side-cavity nests in those provinces due to prevalence of large diameter trees.

Broken-top trees with overhead secondary growth cover may also be used more by owls for nesting than broken-top trees without secondary cover [17]. Indeed, even in the Eastern Cascades Province where platform nests were the most prevalent (generally in trees < 150 y old) NSOs are more likely to reuse cavity nests than non-cavity nests, suggesting selection for cavity nest sites [16, 18, 21, 43]. Side-cavity and top-cavity nests characterized the west side of the Cascades Range (86% of nests) and the trees were larger than trees with platform nests. Side-cavity and top-cavity nests in Douglas-fir trees were similar in size, but western red cedar and western hemlock trees with side-cavity nests were larger than nest trees with broken-tops, re-emphasizing that tree size is a factor for side-cavity nests.

On the Olympic Peninsula, old forests are largely in the federal reserves in the core area of the Olympic National Park and portions of the Olympic National Forest, and were surmised to likely protect enough old forest for a persistent population of NSOs [44]. The high growing season precipitation in a relatively moderate year-round climate, with relatively cool summers may enhance tree establishment and help develop large trees such as those occurring in the Olympic Rain Forest, and fires and destructive wind events are sufficiently rare, particularly in the western region of the Olympic Peninsula [30, 45, 46]. If growth rates are enhanced, habitat can reestablish more quickly [46], and development of younger stands outside federal reserves if allowed to progress through successional stages will contribute to more suitable conditions for NSOs [44]. For example, abundance of large-diameter snags with adequate canopy closure in “submature” forests are important habitats used by NSOs on the western Olympic Peninsula [47].

Although our dataset was larger than many of the NSO nest tree studies published in the last 30 years, our findings largely support other studies cited throughout this work. For example, in studies ranging from the eastern Cascades in Washington to northwestern California, nests were largely in living trees and in Douglas-fir trees [8, 15–20]. Most studies reported a majority of nests in top cavities or side cavities, on the Olympic Peninsula [15, 17], in southwestern Oregon [15], and in northwestern California [19, 20]. Platform nests were prevalent in xeric forests on the east slope of the Cascades Range [16, 18]. Like our findings, these studies indicated that nest trees were large (S2 Table). Top and side-cavity nest trees were largest and platform nest trees were smaller, especially in the eastern Cascades [8, 15–17, 19, 20].

Many of the platform nests observed by field crews had bases of dwarf mistletoe infections and were most frequently found in regions with higher prevalence of Douglas-fir dwarf mistletoe (*A*. *douglasii*) [48]. The effects of dwarf mistletoe on trees increases with severity of infection that include altered tree form, reduced vigor, increased susceptibility to other disturbance agents, and with extreme infections, growth rate, top-killing, and death [48]. These infections benefit a wide range of wildlife species’ habitat and are used for roosting, foraging, food caching, and nesting by small mammals, (e.g., *Arborimus* spp., *Tamiasciurus* spp., *Tamias* spp., *Glaucomys* spp., and *Neotoma* spp.), many small birds, and other owl species [49–52]; including cavity nests resulting from infected decadent trees [53]. These mistletoe infections appear to be absent or uncommon in coastal mountain ranges in Oregon and Washington, suggesting availability of these structures is the primary driver in use for nesting by NSOs. Where dwarf mistletoe occurs, infections are typically more prevalent in the largest trees within the stand [54], but we observed platforms in smaller trees than trees with cavity nests. Mistletoe infections may accelerate the suitability of large trees for nesting by NSOs.

For this analysis we used the common set of variables available for all study areas and because independent habitat characterizations of study areas resulted in differences in the measured forest attributes [55]. Fortunately, data in common that we summarized were likely the most important variables regarding nest trees. Additional factors that may predict nest tree use are elevation, aspect, and slope. However, with suitable nest tree and prey availability within a closed-canopy forest such features are likely minor or of inconclusive importance [8, 19, 56]. Protection from storms, optimal energy balance, or predator avoidance may best explain nest characteristics [16–17, 57].

Our study suggests that NSOs require large stable platforms or cavities for nesting and old forest is a critical resource for NSO persistence because trees with large cavities and/or heavy infestations of dwarf mistletoes are typically most abundant in forests comprised of large old trees [32, 43, 58]. Many of the trees in our study areas may be at least 700 years old [16, 20]. Managing for the retention of such forests and for their replacement is a significant challenge for land managers, especially in the face of climate change and an increasing human population, but will likely be required for persistence of viable NSO populations.

## Supporting information

S1 Table Nest trees of northern spotted owls subdivided by tree decomposition stage, nest type and study area. (PDF)

S2 Table Comparison of selected measures of nest trees of northern spotted owls from studies from the Olympic Peninsula to northern California, USA. (PDF)

S3 Table Data on nest trees of northern spotted owls in five demographic study areas in Washington and western Oregon USA 1985–2013. (PDF)
