# Supplementary material for: Nest trees of northern spotted owls (Strix occidentalis caurina) in Washington and Oregon, USA
Source: PLoS One. 2018 May 31;13(5):e0197887. doi: 10.1371/journal.pone.0197887 (PMC5979023; doi:10.1371/journal.pone.0197887)
Supplement: S1 Table — (PDF) [file pone.0197887.s001.pdf]

S1 Table. Percentages and 95% CIs of northern spotted owl nest trees in Oregon and Washington, USA, subdivided by tree decomposition stage, nest type and study area.

	OLY E	OLY W	OLY	COA	TYE	KLA	CLE
Alive trees (1–2)							
Platform	15.3 (8–27)	2.8 (1–8)	7.2 (4–12)	7.1 (5–10)	8.3 (6–11)	32.0 (27–37)	88.5 (83–92)
Side cavity	23.7 (15–36)	61.7 (52–70)	48.2 (41–56)	15.1 (12–19)	14.5 (12–18)	8.5 (6–12)	3.3 (2–7)
Top cavity	23.7 (15–36)	18.7 (12–27)	20.5 (15–27)	64.6 (60–69)	67.1 (63–71)	52.5 (47–58)	2.2 (1–6)
Total	62.7 (50–74)	83.2 (75–89)	75.9 (69–82)	86.8 (83–90)	89.9 (87–92)	92.9 (90–95)	94.0 (90–97)
Dead trees (stages 3–7)							
Platform	0.0 (0–6)	0.9 (<1–5)	0.6 (<1–3)	0.0 (0–1)	0.0 (0–1)	0.0 (0–1)	2.7 (1–6)
Side cavity	11.9 (623)	13.1 (8–21)	12.7 (8–19)	7.3 (5–10)	2.7 (2–4)	3.6 (2–6)	1.1 (<1–4)
Top cavity	25.4 (16–38)	2.8 (1–8)	10.8 (7–17)	5.9 (4–9)	7.4 (6–10)	3.6 (2–6)	2.2 (1–6)
	OLY E	OLY W	OLY	COA	TYE	KLA	CLE

Total	37.3 (26–50)	16.8 (11–25)	24.1 (18–31)	13.2 (10–17)	10.1 (8–13)	7.1 (5–10)	6.0 (3–11)
