# Supplementary material for: Nest trees of northern spotted owls (Strix occidentalis caurina) in Washington and Oregon, USA
Source: PLoS One. 2018 May 31;13(5):e0197887. doi: 10.1371/journal.pone.0197887 (PMC5979023; doi:10.1371/journal.pone.0197887)
Supplement: S2 Table — (PDF) [file pone.0197887.s002.pdf]

S2 Table. Percentages composition with 95% CIs of Douglas-fir species, nest type and alive nest trees (A); and diameter at breast height (DBH) and total height with 95% CIs (B) for northern spotted owl nest trees from the Olympic Peninsula to northern California, USA.

A

Study area	Douglas-fir	Top Cavity	Side Cavity	Platform	Alive
W. Cascades, Oregon[a]	87			7	97
E. Cascades, Washington[b]	91.8	5.9	10.6	83.5	88.2
CI	84–96	3–13	6–19	74–90	80–94
E. Cascades, Washington[c]	89.6	4.7	5.1	90.2	--
CI	85–93	3–8	3–8	86–93	--
Olympic Peninsula[d]	31.3	37.1	53.4	9.5	77.6
CI	24–40	29–46	44–62	5–16	69–84
Olympic Peninsula[a]	--	--	67	7	73
W. Oregon and E. Cascades, Oregon[e]	87.2	55.3	8.5	36.2	95.7

CI	75–94	41–69	3–20	24–51	86–99
Coast Range, Oregon[a]	93	--	--	7	83
Klamath Mountains, Oregon[a]	83	--	--	40	90
Northwestern California[f]	82.6	59.4	20.3	20.3	--
CI	72–90	48–70	13–31	13–31	--
Northwestern California[g]	26.7	23.7	10.2	66.1	90.0

[a] Hershey and others (1998).

[b] Buchanan and others (1993).

[c] Sovern and others (2011).

[d] Forsman and Giese (1997).

[e] Forsman and others (1984).

[f] LaHaye and Gutiérrez (1999).

[g] Folliard (1993).

S2 Table

B

	DBH (cm)			Total height (m)		
Study area	Side	Top	Platform	Side	Top	Platform
E. Cascades, Washington [a]	--	94.7	65.8 [h]	--	31.0[h]	--
CI	--	83–107	63–69		29–33	--
Olympic Peninsula [b]	136.6 [h]	--	--	40.6 [h]	--	--
CI	125–148	--	--	38–43	--	--
Olympic Peninsula [c]	147.0 [h]	--	--	45 [h,j]	--	--
CI	99–195			39–51	--	--
W. Oregon and E. Cascades, Oregon [d]	--	135[g]	106	38.1	42.0	--
CI	--	123–147	83–129	33–43	35–49	--
W. Cascades, Oregon [c]	--	138.1[h]	--	--	45[h,j]	--
CI		123–153	--	--	41–49	--

Coast Range, Oregon [c]		154.3[h]	120.0[i]	--	--	48[i,j]
CI		139–170	90–150	--	--	44–52
Klamath Mountains, Oregon[c]	--	122.2[h]	--	--	41[h,j]	--
CI	--	107–137	--	--	37–45	--
Northwestern California [e]	157	138	119	43	39	43
CI	151–163	135–141	114–124	41–45	38–40	40–46
Northwestern California [f]			125.7[h]			38.4[h]
CI			110–141			35–42

[a] Buchanan and others (1993).

[b] Forsman and Giese (1997).

[c] Hershey and others (1998).

[d] Forsman and others (1984).

[e] LaHaye and Gutiérrez (1999).

[f] Folliard (1993).

[g] Top and side cavity nests combined.

[h] All trees across sub-categories combined.

[i]Data for all platform nest trees across multiple study areas (see cited source).

[j]Alive trees only (see source for dead tree data).
