# Supplementary material for: Nest trees of northern spotted owls (Strix occidentalis caurina) in Washington and Oregon, USA
Source: PLoS One. 2018 May 31;13(5):e0197887. doi: 10.1371/journal.pone.0197887 (PMC5979023; doi:10.1371/journal.pone.0197887)
Supplement: S3 Table — (PDF) [file pone.0197887.s003.pdf]

S3 Table. Data on nest trees of northern spotted owls in five demographic study areas in Washington and western Oregon USA 1985--2013.

	DSA	State	Nest	Tree	DBH	Stage	Ht
1	OLY E	1	TC	PSME	70	5	7
2	OLY E	1	TC	PSME	119	2	9
3	OLY W	1	TC	PSME	162	6	10
4	OLY E	1	TC	PSME	79	5	11
5	OLY W	1	TC	TSHE	87	4	12
6	OLY E	1	PL	PSME	30	2	13
7	OLY W	1	SC	TSHE	95	4	13
8	OLY E	1	SC	TSHE	49	2	14
9	OLY E	1	SC	PSME	63	5	14
10	OLY E	1	SC	THPL	113	4	15
11	OLY E	1	TC	PIMO	78	4	15
12	OLY E	1	TC	PSME	92	5	15
13	OLY E	1	TC	PSME	68	4	16
14	OLY E	1	TC	TSHE	104	5	16
15	OLY E	1	TC	TSHE	41	5	16
16	OLY W	1	SC	PSME	68	4	18
17	OLY E	1	SC	PSME	64	4	18
18	OLY W	1	SC	PSME	146	2	19
19	OLY W	1	TC	TSHE	59	5	19
20	OLY W	1	SC	TSHE	83	3	20
21	OLY W	1	SC	TSHE	60	4	20
22	OLY E	1	TC	THPL	62	4	21
23	OLY W	1	SC	TSHE	80	3	22
24	OLY W	1	SC	TSHE	106	4	22
25	OLY W	1	SC	PSME	128	6	23
26	OLY E	1	TC	ABAM	101	2	25
27	OLY E	1	SC	PSME	50	2	26
28	OLY E	1	TC	PSME	139	3	26
29	OLY W	1	SC	THPL	121	4	26
30	OLY E	1	PL	PSME	63	1	27
31	OLY E	1	SC	ABAM	100	2	28
32	OLY E	1	SC	THPL	222	5	28
33	OLY W	1	SC	TSHE	53	2	29
34	OLY E	1	TC	PSME	129	3	29
35	OLY E	1	SC	TSHE	83	3	29
36	OLY E	1	SC	TSHE	92	1	30
37	OLY W	1	SC	TSHE	111	4	30
38	OLY W	1	SC	TSHE	100	5	30
39	OLY W	1	TC	PSME	122	2	31
40	OLY E	1	SC	PSME	119	2	32
41	OLY E	1	PL	TSHE	41	2	32
42	OLY W	1	TC	TSHE	59	2	33
43	OLY W	1	SC	TSHE	111	1	34
44	OLY E	1	PL	PSME	63	1	34
45	OLY W	1	SC	TSHE	60	2	34
46	OLY E	1	TC	TSHE	59	2	34
47	OLY E	1	TC	TSHE	78	3	34
48	OLY E	1	SC	TSHE	132	4	34
49	OLY E	1	SC	PSME	128	5	34
50	OLY E	1	TC	PSME	109	2	35
51	OLY W	1	TC	PSME	96	2	35
52	OLY W	1	TC	PSME	90	2	35
53	OLY E	1	TC	TSHE	89	2	35
54	OLY W	1	SC	THPL	209	2	36
55	OLY W	1	TC	PISI	151	2	36
56	OLY W	1	SC	TSHE	92	2	36
57	OLY W	1	TC	TSHE	85	2	36
58	OLY W	1	SC	THPL	155	1	37
59	OLY E	1	PL	PSME	80	1	37
60	OLY E	1	PL	TSHE	71	1	37
61	OLY W	1	SC	TSHE	116	2	37

62	OLY W	1	SC	TSHE	98	2	37
63	OLY W	1	SC	TSHE	97	2	37
64	OLY W	1	SC	THPL	182	4	37
65	OLY E	1	SC	PSME	165	1	38
66	OLY E	1	TC	PSME	114	2	38
67	OLY W	1	SC	TSHE	83	2	38
68	OLY E	1	SC	PSME	80	2	38
69	OLY E	1	TC	THPL	196	2	39
70	OLY W	1	SC	TSHE	163	2	39
71	OLY W	1	SC	TSHE	102	2	39
72	OLY E	1	TC	PSME	80	3	39
73	OLY W	1	PL	ABGR	77	1	40
74	OLY W	1	SC	THPL	238	2	40
75	OLY W	1	SC	THPL	170	2	40
76	OLY W	1	TC	TSHE	115	2	40
77	OLY W	1	SC	THPL	143	2	41
78	OLY E	1	TC	PSME	125	2	41
79	OLY E	1	SC	TSHE	90	2	41
80	OLY E	1	SC	TSME	195	1	42
81	OLY E	1	PL	PSME	176	1	42
82	OLY W	1	SC	TSHE	93	1	42
83	OLY W	1	SC	THPL	215	2	42
84	OLY W	1	TC	THPL	203	2	42
85	OLY W	1	SC	THPL	168	2	42
86	OLY E	1	TC	THPL	155	2	42
87	OLY W	1	SC	THPL	134	2	42
88	OLY E	1	TC	PSME	180	5	42
89	OLY E	1	TC	PSME	89	3	43
90	OLY W	1	TC	PSME	204	1	44
91	OLY W	1	SC	TSHE	105	2	44
92	OLY E	1	SC	PSME	92	2	44
93	OLY W	1	PL	TSHE	84	1	45
94	OLY W	1	SC	THPL	193	2	45
95	OLY W	1	SC	TSHE	126	2	45
96	OLY E	1	TC	TSHE	100	2	45
97	OLY W	1	SC	TSHE	143	3	45
98	OLY W	1	SC	TSHE	122	1	46
99	OLY W	1	SC	THPL	240	2	46
100	OLY E	1	SC	PSME	171	2	46
101	OLY E	1	TC	PSME	183	3	46
102	OLY W	1	SC	THPL	300	2	47
103	OLY W	1	SC	THPL	180	2	47
104	OLY W	1	SC	THPL	175	2	47
105	OLY E	1	SC	TSHE	132	2	47
106	OLY W	1	SC	TSHE	106	2	47
107	OLY E	1	SC	PSME	98	2	47
108	OLY E	1	PL	PSME	74	2	47
109	OLY W	1	SC	THPL	159	3	47
110	OLY W	1	SC	THPL	278	2	48
111	OLY W	1	TC	THPL	218	2	48
112	OLY W	1	TC	THPL	162	2	48
113	OLY W	1	SC	TSHE	148	2	48
114	OLY W	1	SC	TSHE	113	2	48
115	OLY W	1	TC	THPL	108	2	48
116	OLY W	1	SC	TSHE	101	2	48
117	OLY W	1	SC	THPL	165	2	49
118	OLY W	1	TC	TSHE	105	2	49
119	OLY W	1	SC	TSHE	129	1	50
120	OLY E	1	PL	ABGR	100	1	50
121	OLY W	1	SC	THPL	300	2	50
122	OLY E	1	TC	PSME	174	2	50
123	OLY E	1	TC	PSME	158	2	50
124	OLY W	1	SC	TSHE	119	2	50
125	OLY W	1	TC	TSHE	106	2	50
126	OLY W	1	SC	THPL	360	2	51
127	OLY W	1	SC	THPL	189	2	51

128	OLY W	1	SC	ABAM	133	2	51
129	OLY W	1	TC	TSHE	125	2	51
130	OLY W	1	SC	TSHE	120	1	52
131	OLY W	1	SC	PSME	93	1	52
132	OLY W	1	SC	THPL	280	2	52
133	OLY W	1	TC	THPL	215	2	52
134	OLY E	1	SC	THPL	179	2	52
135	OLY E	1	TC	PSME	116	2	52
136	OLY W	1	SC	TSHE	76	2	52
137	OLY W	1	SC	THPL	379	2	53
138	OLY W	1	SC	THPL	314	2	53
139	OLY W	1	SC	THPL	313	2	53
140	OLY W	1	SC	THPL	285	2	53
141	OLY W	1	SC	THPL	150	2	53
142	OLY W	1	SC	TSHE	150	2	53
143	OLY W	1	SC	TSHE	128	2	53
144	OLY W	1	SC	THPL	105	2	53
145	OLY W	1	SC	TSHE	105	2	53
146	OLY W	1	PL	THPL	198	3	53
147	OLY W	1	SC	THPL	222	2	54
148	OLY W	1	TC	PSME	183	2	54
149	OLY W	1	SC	THPL	98	4	54
150	OLY E	1	TC	PSME	199	2	55
151	OLY W	1	SC	TSHE	154	2	55
152	OLY W	1	TC	PSME	86	2	56
153	OLY W	1	SC	THPL	272	2	57
154	OLY W	1	SC	TSHE	141	1	58
155	OLY W	1	SC	THPL	320	2	58
156	OLY W	1	SC	THPL	300	2	58
157	OLY W	1	SC	PSME	233	2	59
158	OLY W	1	SC	THPL	205	2	59
159	OLY W	1	PL	PSME	175	1	60
160	OLY W	1	SC	THPL	267	2	61
161	OLY W	1	SC	THPL	160	2	61
162	OLY W	1	TC	THPL	232	1	65
163	OLY W	1	SC	TSHE	112	1	65
164	OLY W	1	SC	THPL	268	2	67
165	OLY W	1	TC	PSME	136	2	68
166	OLY W	1	SC	PSME	140	2	74
167	CLE	1	PL	PSME	62		41
168	CLE	1	PL	PSME	29		22
169	CLE	1	PL	PSME	74		46
170	CLE	1	PL	PSME	89		35
171	CLE	1	PL	PSME	67		41
172	CLE	1	PL	PSME	86		34
173	CLE	1	PL	PSME	83		34
174	CLE	1	PL	PSME	75		41
175	CLE	1	PL	PSME	51		30
176	CLE	1	PL	PSME	73		38
177	CLE	1	PL	PSME	90		31
178	CLE	1	PL	PSME	55		22
179	CLE	1	TC	PSME	122		23
180	CLE	1	PL	PSME	29		24
181	CLE	1	PL	PSME	58		33
182	CLE	1	PL	PSME	38		24
183	CLE	1	PL	PSME	39		24
184	CLE	1	PL	PSME	42		24
185	CLE	1	PL	PSME	46		30
186	CLE	1	TC	PSME	173		48
187	CLE	1	TC	PSME	163		30
188	CLE	1	SC	TSHE	138		30
189	CLE	1	PL	TSHE	85		43
190	CLE	1	PL	PSME	86		27
191	CLE	1	SC	THPL	145		32
192	CLE	1	PL	TSHE	48		27
193	CLE	1	PL	PSME	75		23

194	CLE	1	PL	PSME	42	30
195	CLE	1	PL	PSME	29	17
196	CLE	1	PL	PSME	80	39
197	CLE	1	PL	PSME	56	36
198	CLE	1	PL	PSME	72	40
199	CLE	1	PL	PSME	70	45
200	CLE	1	PL	PSME	58	37
201	CLE	1	PL	PSME	50	29
202	CLE	1	PL	PSME	73	43
203	CLE	1	PL	PSME	83	30
204	CLE	1	PL	PSME	36	25
205	CLE	1	PL	PSME	47	27
206	CLE	1	PL	PSME	62	30
207	CLE	1	SC	TSHE	125	33
208	CLE	1	TC	THPL	150	33
209	CLE	1	SC	PSME	141	36
210	CLE	1	SC	TSHE	102	27
211	CLE	1	PL	PSME	91	23
212	CLE	1	PL	PSME	58	35
213	CLE	1	PL	PSME	58	38
214	CLE	1	PL	PSME	103	48
215	CLE	1	PL	PSME	55	30
216	CLE	1	PL	PSME	46	35
217	CLE	1	SC	PSME	115	29
218	CLE	1	PL	PSME	60	33
219	CLE	1	PL	PSME	90	32
220	CLE	1	SC	PSME	95	30
221	CLE	1	PL	PSME	72	29
222	CLE	1	PL	PSME	67	29
223	CLE	1	PL	PSME	21	19
224	CLE	1	TC	PSME	92	21
225	CLE	1	PL	PSME	51	38
226	CLE	1	PL	PSME	62	36
227	CLE	1	PL	ABGR	54	32
228	CLE	1	PL	PSME	70	38
229	CLE	1	PL	PSME	62	39
230	CLE	1	PL	PSME	74	42
231	CLE	1	PL	PSME	49	36
232	CLE	1	PL	PSME	54	31
233	CLE	1	PL	PSME	56	37
234	CLE	1	PL	PSME	54	30
235	CLE	1	PL	PSME	90	47
236	CLE	1	PL	PSME	67	45
237	CLE	1	PL	PSME	41	30
238	CLE	1	PL	PSME	35	24
239	CLE	1	PL	PSME	77	38
240	CLE	1	PL	PSME	60	25
241	CLE	1	PL	PSME	68	40
242	CLE	1	PL	PSME	38	29
243	CLE	1	PL	PSME	47	30
244	CLE	1	PL	PSME	60	35
245	CLE	1	PL	PSME	58	33
246	CLE	1	PL	PSME	59	33
247	CLE	1	PL	ABGR	52	28
248	CLE	1	PL	PSME	48	33
249	CLE	1	PL	PSME	33	32
250	CLE	1	PL	PSME	71	41
251	CLE	1	PL	PSME	66	27
252	CLE	1	PL	PSME	29	22
253	CLE	1	PL	PSME	39	29
254	CLE	1	PL	PSME	35	31
255	CLE	1	PL	PSME	37	23
256	CLE	1	PL	PSME	61	38
257	CLE	1	PL	PSME	100	36
258	CLE	1	PL	PSME	56	24
259	CLE	1	PL	PSME	52	27

260	CLE	1	PL	PSME	97	36
261	CLE	1	PL	PIPO	54	32
262	CLE	1	PL	PSME	50	28
263	CLE	1	PL	PSME	43	25
264	CLE	1	PL	PSME	54	32
265	CLE	1	PL	PSME	87	47
266	CLE	1	PL	PSME	42	24
267	CLE	1	TC	PSME	91	14
268	CLE	1	PL	PSME	66	33
269	CLE	1	PL	PSME	31	27
270	CLE	1	PL	PSME	82	29
271	CLE	1	PL	PSME	61	32
272	CLE	1	PL	PSME	58	30
273	CLE	1	PL	PSME	56	34
274	CLE	1	PL	PSME	48	32
275	CLE	1	PL	ABGR	49	35
276	CLE	1	PL	PSME	31	23
277	CLE	1	PL	PSME	71	30
278	CLE	1	PL	PSME	51	27
279	CLE	1	PL	PSME	29	19
280	CLE	1	PL	PSME	83	28
281	CLE	1	PL	PSME	50	30
282	CLE	1	PL	PSME	41	34
283	CLE	1	PL	PSME	34	25
284	CLE	1	PL	PSME	83	40
285	CLE	1	PL	PSME	56	25
286	CLE	1	PL	PSME	35	24
287	CLE	1	PL	PSME	64	31
288	CLE	1	PL	PSME	59	34
289	CLE	1	PL	ABGR	82	34
290	CLE	1	PL	PSME	75	41
291	CLE	1	PL	PSME	48	28
292	CLE	1	PL	PSME	54	31
293	CLE	1	PL	PSME	58	36
294	CLE	1	PL	PSME	49	37
295	CLE	1	PL	PSME	51	32
296	CLE	1	PL	PSME	50	30
297	CLE	1	PL	PSME	96	32
298	CLE	1	PL	PSME	82	38
299	CLE	1	PL	PSME	21	29
300	CLE	1	PL	PSME	51	39
301	CLE	1	PL	PSME	24	23
302	CLE	1	PL	PSME	68	40
303	CLE	1	PL	PSME	73	23
304	CLE	1	PL	PSME	67	40
305	CLE	1	PL	PSME	72	35
306	CLE	1	PL	PSME	68	35
307	CLE	1	PL	PSME	68	46
308	CLE	1	PL	PSME	73	33
309	CLE	1	PL	PSME	45	27
310	CLE	1	PL	PSME	56	24
311	CLE	1	PL	PSME	56	32
312	CLE	1	PL	PSME	42	28
313	CLE	1	PL	PSME	39	39
314	CLE	1	PL	PSME	40	24
315	CLE	1	PL	PSME	36	29
316	CLE	1	PL	PSME	49	33
317	CLE	1	PL	PSME	54	31
318	CLE	1	PL	PSME	59	34
319	CLE	1	PL	PSME	46	39
320	CLE	1	PL	PSME	99	28
321	CLE	1	PL	LAOC	34	31
322	CLE	1	PL	PSME	72	32
323	CLE	1	PL	PSME	62	30
324	CLE	1	TC	PSME	94	8
325	CLE	1	PL	PSME	41	30

326	CLE	1	TC	PIPO	67		36
327	CLE	1	PL	PSME	76		39
328	CLE	1	PL	PSME	59		27
329	CLE	1	PL	PSME	81		35
330	CLE	1	PL	PSME	87		33
331	CLE	1	PL	PSME	59		34
332	CLE	1	PL	PSME	49		37
333	CLE	1	PL	PSME	47		30
334	CLE	1	PL	PSME	31		25
335	CLE	1	PL	PSME	65		33
336	CLE	1	PL	PSME	42		25
337	CLE	1	PL	PSME	64		48
338	CLE	1	PL	PSME	33		29
339	CLE	1	PL	PSME	71		41
340	CLE	1	PL	PSME	57		36
341	CLE	1	PL	PSME	58		34
342	CLE	1	PL	PSME	45		32
343	CLE	1	PL	PSME	37		31
344	CLE	1	SC	THPL	99		25
345	CLE	1	PL	PSME	81		35
346	CLE	1	PL	PSME	103		40
347	CLE	1	PL	PSME	37		35
348	CLE	1	PL	PSME	57		28
349	COA	2	TC	PSME	142	2	44
350	COA	2	TC	PSME	163	3	13
351	COA	2	SC	PSME	130	2	30
352	COA	2	TC	PSME	120	2	24
353	COA	2	TC	PSME	168	2	61
354	COA	2	TC	PSME	197	2	54
355	COA	2	TC	PSME	190	2	62
356	COA	2	TC	PSME	177	2	60
357	COA	2	SC	THPL	152	2	25
358	COA	2	TC	PSME	132	2	53
359	COA	2	TC	PSME	179	2	64
360	COA	2	TC	PSME	189	2	65
361	COA	2	TC	PSME	137	2	44
362	COA	2	TC	PSME	138	2	27
363	COA	2	TC	PSME	131	2	41
364	COA	2	TC	PSME	132	2	33
365	COA	2	TC	PSME	107	2	28
366	COA	2	TC	PSME	149	2	52
367	COA	2	TC	PSME	121	2	42
368	COA	2	TC	PSME	196	2	59
369	COA	2	TC	PSME	109	2	45
370	COA	2	TC	PSME	180	2	37
371	COA	2	TC	PSME	136	2	44
372	COA	2	TC	PSME	171	2	57
373	COA	2	TC	PSME	135	2	42
374	COA	2	TC	PSME	94	2	37
375	COA	2	TC	PSME	142	2	53
376	COA	2	TC	PSME	135	2	24
377	COA	2	SC	TSHE	83	2	39
378	COA	2	SC	THPL	168	1	40
379	COA	2	TC	PSME	151	2	54
380	COA	2	TC	PSME	127	2	44
381	COA	2	TC	PSME	213	2	47
382	COA	2	SC	PSME	122	2	36
383	COA	2	TC	PSME	148	2	25
384	COA	2	TC	PSME	91	2	54
385	COA	2	TC	PSME	102	2	38
386	COA	2	TC	PSME	188	2	40
387	COA	2	TC	PSME	137	2	52
388	COA	2	TC	PSME	144	2	38
389	COA	2	PL	PSME	149	1	52
390	COA	2	SC	PSME	88	6	27
391	COA	2	TC	PSME	143	2	58

392	COA	2	TC	PSME	117	2	46
393	COA	2	TC	PSME	150	2	47
394	COA	2	TC	PSME	171	2	50
395	COA	2	TC	PSME	113	2	40
396	COA	2	SC	PSME	188	2	40
397	COA	2	TC	PSME	109	2	42
398	COA	2	TC	PSME	142	2	45
399	COA	2	TC	PSME	99	2	23
400	COA	2	TC	PSME	153	2	40
401	COA	2	TC	PSME	170	2	51
402	COA	2	TC	PSME	130	2	33
403	COA	2	TC	PSME	159	2	35
404	COA	2	SC	PSME	93	1	56
405	COA	2	TC	PSME	109	2	43
406	COA	2	TC	PSME	180	6	13
407	COA	2	TC	PSME	145	2	33
408	COA	2	SC	PSME	118	2	37
409	COA	2	TC	PSME	161	1	52
410	COA	2	SC	THPL	151	2	46
411	COA	2	SC	PSME	170	2	34
412	COA	2	TC	PSME	155	2	18
413	COA	2	SC	PSME	183	2	33
414	COA	2	SC	PSME	181	2	56
415	COA	2	TC	PSME	193	2	47
416	COA	2	SC	PSME	167	1	56
417	COA	2	TC	PSME	111	2	34
418	COA	2	SC	PSME	144	2	42
419	COA	2	TC	PSME	163	2	42
420	COA	2	SC	PSME	123	2	26
421	COA	2	SC	PSME	130	1	52
422	COA	2	TC	PSME	155	2	37
423	COA	2	TC	PSME	138	2	36
424	COA	2	TC	PSME	170	2	66
425	COA	2	SC	PSME	147	2	38
426	COA	2	TC	PSME	88	2	38
427	COA	2	TC	THPL	142	2	40
428	COA	2	TC	PSME	135	2	55
429	COA	2	SC	PSME	107	2	20
430	COA	2	TC	PSME	115	2	48
431	COA	2	TC	PSME	180	2	60
432	COA	2	TC	PSME	146	6	13
433	COA	2	TC	PSME	148	2	32
434	COA	2	SC	PSME	145	2	40
435	COA	2	SC	PSME	109	7	20
436	COA	2	TC	PSME	166	6	22
437	COA	2	TC	PSME	115	2	27
438	COA	2	TC	THPL	118	6	17
439	COA	2	SC	PSME	61	4	16
440	COA	2	SC	PSME	106	4	19
441	COA	2	TC	ACMA	104	2	39
442	COA	2	TC	PSME	156	2	49
443	COA	2	TC	PSME	110	2	26
444	COA	2	PL	PSME	192	2	64
445	COA	2	TC	PSME	154	2	52
446	COA	2	TC	PSME	160	2	56
447	COA	2	PL	PSME	140	1	70
448	COA	2	TC	PSME	187	2	44
449	COA	2	TC	PSME	107	2	49
450	COA	2	TC	PSME	181	2	53
451	COA	2	TC	PSME	109	2	37
452	COA	2	TC	PSME	188	2	65
453	COA	2	TC	PSME	213	2	52
454	COA	2	TC	PSME	142	2	48
455	COA	2	PL	PSME	131	1	42
456	COA	2	SC	ACMA	85	1	29
457	COA	2	SC	PSME	105	7	19

458	COA	2	SC	PSME	107	4	24
459	COA	2	TC	PSME	155	2	28
460	COA	2	TC	PSME	168	2	35
461	COA	2	TC	ACMA	102	2	26
462	COA	2	TC	PSME	104	6	22
463	COA	2	TC	PSME	102	7	18
464	COA	2	TC	PSME	164	1	55
465	COA	2	TC	PSME	147	2	55
466	COA	2	SC	THPL	96	6	16
467	COA	2	TC	PSME	141	2	43
468	COA	2	SC	PSME	163	1	50
469	COA	2	TC	PSME	186	2	64
470	COA	2	TC	PSME	122	2	32
471	COA	2	TC	THPL	124	2	39
472	COA	2	TC	PSME	147	2	37
473	COA	2	TC	PSME	114	2	39
474	COA	2	TC	PSME	182	2	44
475	COA	2	PL	PSME	79	1	39
476	COA	2	TC	PSME	160	2	46
477	COA	2	TC	PSME	62	2	6
478	COA	2	TC	PSME	146	2	33
479	COA	2	TC	PSME	133	2	47
480	COA	2	TC	PSME	164	3	28
481	COA	2	SC	THPL	147	6	18
482	COA	2	SC	PSME	139	6	41
483	COA	2	TC	PSME	122	2	38
484	COA	2	PL	CADE	48	1	28
485	COA	2	TC	PSME	127	2	29
486	COA	2	PL	PSME	98	2	34
487	COA	2	TC	PSME	110	2	42
488	COA	2	TC	PSME	109	2	24
489	COA	2	PL	PSME	109	1	45
490	COA	2	SC	PSME	145	2	52
491	COA	2	TC	PSME	127	2	38
492	COA	2	PL	PSME	141	1	29
493	COA	2	SC	PSME	187	2	56
494	COA	2	TC	PSME	142	2	12
495	COA	2	TC	PSME	175	2	44
496	COA	2	PL	ABGR	93	1	37
497	COA	2	SC	TSHE	114	3	44
498	COA	2	TC	PSME	120	2	47
499	COA	2	TC	PSME	164	2	42
500	COA	2	PL	PSME	216	1	30
501	COA	2	PL	PSME	140	1	50
502	COA	2	TC	PSME	182	2	44
503	COA	2	TC	PSME	106	2	45
504	COA	2	TC	PSME	109	2	42
505	COA	2	TC	PSME	124	2	26
506	COA	2	TC	PSME	105	2	19
507	COA	2	SC	THPL	89	6	17
508	COA	2	TC	UNKN	114	6	14
509	COA	2	TC	PSME	141	2	41
510	COA	2	TC	PSME	172	2	50
511	COA	2	SC	ARME	67	2	20
512	COA	2	TC	THPL	168	2	47
513	COA	2	TC	PSME	142	2	33
514	COA	2	TC	PSME	155	2	47
515	COA	2	TC	PSME	104	2	30
516	COA	2	TC	PSME	150	2	34
517	COA	2	TC	PSME	161	2	51
518	COA	2	SC	PSME	202	2	58
519	COA	2	TC	PSME	134	2	52
520	COA	2	TC	PSME	143	2	56
521	COA	2	TC	PSME	130	2	34
522	COA	2	TC	PSME	142	2	43
523	COA	2	TC	PSME	171	2	54

524	COA	2	SC	PSME	96	6	12
525	COA	2	TC	PSME	161	2	37
526	COA	2	TC	PSME	110	2	31
527	COA	2	TC	PSME	144	2	44
528	COA	2	SC	PSME	159	3	34
529	COA	2	TC	PSME	109	2	35
530	COA	2	TC	PSME	114	2	25
531	COA	2	TC	PSME	154	2	47
532	COA	2	TC	PSME	159	2	44
533	COA	2	PL	PSME	183	2	45
534	COA	2	TC	PSME	127	2	28
535	COA	2	SC	THPL	107	2	41
536	COA	2	TC	PSME	106	2	32
537	COA	2	SC	PSME	138	3	38
538	COA	2	TC	PSME	180	2	30
539	COA	2	SC	PSME	144	2	31
540	COA	2	TC	PSME	132	2	48
541	COA	2	SC	PSME	136	2	45
542	COA	2	TC	PSME	168	2	48
543	COA	2	SC	THPL	130	2	44
544	COA	2	TC	PSME	129	2	48
545	COA	2	SC	PSME	135	2	35
546	COA	2	TC	PSME	147	2	37
547	COA	2	TC	PSME	137	2	32
548	COA	2	PL	THPL	116	1	49
549	COA	2	SC	PSME	189	2	56
550	COA	2	SC	PSME	157	6	30
551	COA	2	TC	PSME	157	2	48
552	COA	2	TC	PSME	125	6	13
553	COA	2	TC	PSME	175	2	46
554	COA	2	TC	PSME	115	2	40
555	COA	2	TC	PSME	154	2	47
556	COA	2	SC	PSME	147	1	60
557	COA	2	SC	PSME	120	7	8
558	COA	2	TC	PSME	144	2	44
559	COA	2	TC	THPL	244	2	45
560	COA	2	SC	PSME	170	2	36
561	COA	2	TC	PSME	123	2	48
562	COA	2	TC	PSME	183	2	48
563	COA	2	TC	PSME	147	2	36
564	COA	2	TC	PSME	139	2	41
565	COA	2	TC	PSME	122	2	29
566	COA	2	SC	PSME	95	7	9
567	COA	2	TC	PSME	120	2	50
568	COA	2	TC	PSME	103	2	38
569	COA	2	PL	PSME	190	1	73
570	COA	2	TC	PSME	150	2	49
571	COA	2	TC	PSME	141	2	35
572	COA	2	TC	PSME	175	2	35
573	COA	2	TC	PSME	113	2	44
574	COA	2	TC	PSME	152	2	61
575	COA	2	TC	PSME	112	2	35
576	COA	2	PL	PSME	138	1	37
577	COA	2	TC	PSME	122	2	40
578	COA	2	TC	PSME	178	2	55
579	COA	2	TC	PSME	191	2	60
580	COA	2	SC	PSME	130	2	40
581	COA	2	TC	PSME	167	2	42
582	COA	2	TC	PSME	140	2	23
583	COA	2	TC	PSME	132	2	19
584	COA	2	TC	PSME	130	2	29
585	COA	2	TC	PSME	108	2	26
586	COA	2	PL	PSME	127	1	47
587	COA	2	SC	PSME	83	2	39
588	COA	2	SC	PSME	61	2	25
589	COA	2	TC	PSME	135	2	46

590	COA	2	TC	PSME	152	2	40
591	COA	2	TC	PSME	95	2	28
592	COA	2	TC	PSME	122	2	34
593	COA	2	SC	PSME	164	2	70
594	COA	2	TC	PSME	157	2	45
595	COA	2	TC	PSME	147	2	58
596	COA	2	TC	PSME	190	2	48
597	COA	2	TC	PSME	137	2	49
598	COA	2	TC	PSME	120	2	47
599	COA	2	TC	PSME	159	2	49
600	COA	2	SC	PSME	155	2	46
601	COA	2	TC	PSME	123	2	39
602	COA	2	PL	PSME	83	1	43
603	COA	2	TC	PSME	177	2	49
604	COA	2	TC	PSME	102	6	14
605	COA	2	TC	PSME	94	2	29
606	COA	2	TC	PSME	97	2	20
607	COA	2	TC	PSME	89	2	33
608	COA	2	TC	PSME	141	2	21
609	COA	2	TC	PSME	137	2	35
610	COA	2	TC	PSME	164	2	45
611	COA	2	TC	PSME	137	2	39
612	COA	2	PL	PSME	87	1	48
613	COA	2	TC	PSME	115	2	26
614	COA	2	TC	PSME	130	2	48
615	COA	2	PL	PSME	166	1	48
616	COA	2	TC	PSME	152	2	34
617	COA	2	SC	PSME	198	2	54
618	COA	2	TC	PSME	219	2	38
619	COA	2	TC	PSME	79	2	22
620	COA	2	TC	PSME	126	2	34
621	COA	2	TC	PSME	180	2	45
622	COA	2	TC	PSME	181	2	48
623	COA	2	TC	PSME	153	2	44
624	COA	2	TC	PSME	180	2	53
625	COA	2	TC	PSME	114	6	15
626	COA	2	TC	PSME	155	2	57
627	COA	2	TC	PSME	126	2	52
628	COA	2	PL	PSME	80	2	36
629	COA	2	TC	PSME	107	2	35
630	COA	2	TC	PSME	97	3	20
631	COA	2	TC	PSME	117	2	42
632	COA	2	TC	PSME	190	2	56
633	COA	2	SC	PSME	124	1	43
634	COA	2	TC	PSME	134	2	54
635	COA	2	TC	PSME	113	2	37
636	COA	2	TC	PSME	139	2	38
637	COA	2	TC	PSME	136	2	32
638	COA	2	TC	PSME	120	2	55
639	COA	2	TC	PSME	101	2	37
640	COA	2	TC	PSME	141	2	25
641	COA	2	TC	PSME	145	2	30
642	COA	2	TC	PSME	148	2	46
643	COA	2	TC	PSME	107	2	35
644	COA	2	TC	PSME	137	2	22
645	COA	2	TC	PSME	173	2	40
646	COA	2	TC	PSME	108	2	49
647	COA	2	TC	PSME	185	2	59
648	COA	2	SC	PSME	203	2	69
649	COA	2	TC	PSME	158	2	54
650	COA	2	TC	PSME	161	2	50
651	COA	2	TC	PSME	156	2	46
652	COA	2	TC	PSME	194	2	48
653	COA	2	PL	PSME	175	1	66
654	COA	2	SC	PSME	152	2	48
655	COA	2	TC	PSME	142	2	28

656	COA	2	TC	PSME	183	2	47
657	COA	2	TC	PSME	145	2	33
658	COA	2	TC	PSME	201	6	23
659	COA	2	TC	PSME	197	2	53
660	COA	2	PL	PSME	111	1	47
661	COA	2	TC	PSME	168	2	53
662	COA	2	TC	PSME	114	2	31
663	COA	2	TC	PSME	142	2	40
664	COA	2	TC	PSME	149	2	50
665	COA	2	SC	PSME	142	1	59
666	COA	2	PL	PSME	68	1	45
667	COA	2	PL	PSME	85	1	44
668	COA	2	TC	PSME	127	2	38
669	COA	2	TC	PSME	143	2	44
670	COA	2	TC	UNKN	132	6	14
671	COA	2	TC	PSME	170	2	54
672	COA	2	TC	PSME	165	2	36
673	COA	2	TC	PSME	86	2	16
674	COA	2	TC	PSME	119	2	27
675	COA	2	TC	PSME	109	2	21
676	COA	2	TC	PSME	97	2	46
677	COA	2	TC	PSME	101	2	41
678	COA	2	TC	PSME	120	2	49
679	COA	2	TC	PSME	235	2	58
680	COA	2	SC	THPL	168	6	22
681	COA	2	SC	THPL	273	6	37
682	COA	2	SC	PSME	217	7	35
683	COA	2	SC	THPL	222	2	50
684	COA	2	SC	THPL	286	2	46
685	COA	2	TC	PSME	136	7	8
686	COA	2	SC	THPL	179	2	48
687	COA	2	SC	THPL	213	2	49
688	COA	2	TC	PSME	144	7	18
689	COA	2	SC	ACMA	124	1	30
690	COA	2	SC	THPL	83	6	30
691	COA	2	SC	THPL	183	5	38
692	COA	2	SC	THPL	156	5	49
693	COA	2	PL	PSME	186	2	58
694	COA	2	TC	PSME	115	2	42
695	COA	2	TC	PSME	163	2	47
696	COA	2	SC	PSME	155	7	13
697	COA	2	TC	PSME	148	4	18
698	COA	2	TC	PSME	149	6	25
699	COA	2	TC	PSME	215	7	18
700	COA	2	SC	THPL	233	2	49
701	COA	2	TC	THPL	205	2	50
702	COA	2	SC	THPL	79	5	26
703	COA	2	TC	THPL	126	2	25
704	COA	2	TC	PSME	225	2	48
705	COA	2	TC	PSME	113	2	47
706	COA	2	TC	PSME	214	6	24
707	COA	2	TC	PSME	209	2	56
708	COA	2	TC	PSME	225	2	64
709	COA	2	TC	PSME	200	2	55
710	COA	2	TC	PSME	212	2	48
711	COA	2	TC	PSME	206	2	59
712	COA	2	SC	TSHE	75	3	32
713	COA	2	TC	PSME	143	2	53
714	COA	2	TC	PSME	238	2	49
715	COA	2	TC	PSME	235	2	58
716	COA	2	PL	PSME	151	2	37
717	COA	2	TC	PSME	173	2	47
718	COA	2	PL	PISI	120	1	52
719	COA	2	PL	PISI	139	1	64
720	COA	2	SC	THPL	145	2	35
721	COA	2	TC	PSME	194	2	58

722	COA	2	TC	PSME	179	2	45
723	COA	2	PL	PSME	90	1	52
724	COA	2	TC	PSME	211	2	63
725	COA	2	TC	PSME	161	2	65
726	COA	2	TC	PSME	167	2	53
727	COA	2	SC	PSME	162	6	27
728	COA	2	SC	TSHE	126	1	54
729	COA	2	TC	PSME	214	2	61
730	COA	2	TC	PSME	106	5	20
731	COA	2	TC	PSME	136	2	56
732	COA	2	TC	THPL	151	7	13
733	COA	2	TC	PSME	170	2	61
734	COA	2	SC	PSME	176	2	40
735	COA	2	TC	PSME	124	2	46
736	COA	2	SC	THPL	108	6	28
737	COA	2	SC	THPL	136	5	39
738	COA	2	TC	PSME	138	2	64
739	COA	2	TC	PSME	171	2	64
740	COA	2	TC	PSME	141	2	53
741	COA	2	SC	PSME	148	5	50
742	COA	2	SC	THPL	129	5	42
743	COA	2	SC	THPL	127	2	45
744	COA	2	TC	PSME	156	2	50
745	COA	2	TC	PSME	164	2	54
746	COA	2	TC	PSME	248	2	54
747	COA	2	TC	PSME	161	2	42
748	COA	2	TC	PSME	152	7	11
749	COA	2	SC	PSME	186	2	60
750	COA	2	TC	PSME	140	2	56
751	COA	2	TC	PSME	137	6	19
752	COA	2	TC	PSME	172	2	47
753	COA	2	TC	PSME	179	2	76
754	COA	2	TC	PSME	194	2	60
755	COA	2	TC	PSME	206	2	27
756	COA	2	SC	PSME	183	2	53
757	COA	2	TC	PSME	172	2	45
758	COA	2	PL	THPL	135	1	49
759	COA	2	TC	TSHE	95	2	36
760	COA	2	TC	PSME	130	2	57
761	COA	2	TC	PSME	154	2	49
762	COA	2	SC	PSME	161	1	55
763	COA	2	TC	PSME	233	2	63
764	COA	2	SC	THPL	199	2	37
765	COA	2	TC	PSME	152	2	54
766	COA	2	TC	PSME	146	2	53
767	COA	2	SC	PSME	196	2	48
768	COA	2	TC	PSME	135	2	44
769	COA	2	TC	PSME	56	2	19
770	COA	2	TC	PSME	75	2	37
771	COA	2	TC	PSME	145	2	50
772	COA	2	TC	PSME	212	2	63
773	COA	2	SC	PSME	166	1	56
774	COA	2	SC	PSME	141	6	34
775	COA	2	TC	PSME	172	2	58
776	COA	2	SC	PSME	169	2	49
777	COA	2	SC	PSME	201	2	46
778	COA	2	SC	ACMA	110	2	32
779	COA	2	TC	PSME	179	2	60
780	COA	2	SC	THPL	138	2	43
781	COA	2	TC	PSME	102	2	22
782	COA	2	TC	PSME	169	2	45
783	COA	2	SC	PSME	167	2	45
784	COA	2	SC	PSME	74	2	27
785	COA	2	TC	PSME	116	4	16
786	COA	2	SC	PSME	154	2	38
787	KLA	2	SC	PSME	152	1	18

788	KLA	2	SC	PSME	142	1	18
789	KLA	2	SC	ARME	61	1	21
790	KLA	2	TC	PSME	107	1	22
791	KLA	2	SC	QUKE	58	1	23
792	KLA	2	TC	QUKE	91	1	23
793	KLA	2	PL	PSME	76	1	23
794	KLA	2	PL	PSME	73	1	23
795	KLA	2	PL	PSME	51	1	24
796	KLA	2	PL	PSME	107	1	24
797	KLA	2	TC	PSME	130	1	25
798	KLA	2	PL	PSME	72	1	25
799	KLA	2	TC	PSME	157	1	25
800	KLA	2	TC	PSME	146	1	26
801	KLA	2	TC	PSME	122	1	26
802	KLA	2	SC	PSME	122	1	26
803	KLA	2	TC	PSME	173	1	26
804	KLA	2	PL	ARME	90	1	26
805	KLA	2	PL	PSME	41	1	27
806	KLA	2	TC	PSME	109	1	27
807	KLA	2	PL	PSME	57	1	27
808	KLA	2	PL	CADE	69	1	28
809	KLA	2	TC	PSME	100	1	29
810	KLA	2	SC	PSME	137	1	29
811	KLA	2	PL	PSME	41	1	29
812	KLA	2	TC	PSME	165	1	30
813	KLA	2	PL	PSME	95	1	30
814	KLA	2	TC	PSME	162	1	30
815	KLA	2	PL	PSME	86	1	30
816	KLA	2	SC	PSME	99	1	30
817	KLA	2	SC	PSME	135	1	30
818	KLA	2	TC	PSME	130	1	30
819	KLA	2	TC	PSME	132	1	30
820	KLA	2	SC	PSME	112	1	30
821	KLA	2	SC	PSME	147	1	30
822	KLA	2	PL	PSME	74	1	30
823	KLA	2	PL	CADE	13	1	31
824	KLA	2	PL	CADE	92	1	31
825	KLA	2	PL	PSME	89	1	31
826	KLA	2	SC	PSME	84	1	31
827	KLA	2	TC	PSME	87	1	31
828	KLA	2	PL	CADE	124	1	32
829	KLA	2	TC	PSME	129	1	32
830	KLA	2	SC	PSME	122	1	32
831	KLA	2	PL	PSME	79	1	33
832	KLA	2	PL	PSME	107	1	34
833	KLA	2	TC	PSME	122	1	34
834	KLA	2	TC	PSME	112	1	34
835	KLA	2	TC	PSME	157	1	34
836	KLA	2	TC	PSME	145	1	34
837	KLA	2	PL	PSME	152	1	34
838	KLA	2	SC	PSME	109	1	34
839	KLA	2	PL	PSME	128	1	34
840	KLA	2	PL	PSME	86	1	35
841	KLA	2	TC	PSME	116	1	35
842	KLA	2	PL	PSME	97	1	36
843	KLA	2	PL	PSME	65	1	36
844	KLA	2	PL	PSME	89	1	36
845	KLA	2	PL	PSME	89	1	36
846	KLA	2	PL	PSME	97	1	36
847	KLA	2	PL	PSME	81	1	36
848	KLA	2	PL	PSME	100	1	36
849	KLA	2	PL	PSME	119	1	37
850	KLA	2	TC	PILA	175	1	37
851	KLA	2	PL	PSME	140	1	37
852	KLA	2	PL	PIPO	62	1	37
853	KLA	2	TC	PSME	103	1	37

854	KLA	2	PL	PSME	91	1	37
855	KLA	2	TC	PSME	114	1	37
856	KLA	2	PL	PSME	117	1	37
857	KLA	2	PL	CADE	114	1	37
858	KLA	2	TC	PSME	132	1	37
859	KLA	2	PL	PSME	74	1	37
860	KLA	2	PL	PSME	99	1	37
861	KLA	2	TC	PSME	191	1	37
862	KLA	2	TC	PSME	138	1	38
863	KLA	2	TC	PSME	122	1	38
864	KLA	2	TC	PSME	114	1	39
865	KLA	2	PL	PSME	86	1	39
866	KLA	2	PL	PSME	65	1	40
867	KLA	2	PL	PSME	106	1	40
868	KLA	2	PL	PSME	81	1	40
869	KLA	2	PL	PSME	87	1	40
870	KLA	2	PL	PSME	109	1	40
871	KLA	2	PL	PSME	100	1	40
872	KLA	2	TC	PSME	124	1	40
873	KLA	2	TC	PSME	122	1	40
874	KLA	2	PL	PSME	79	1	40
875	KLA	2	TC	PSME	165	1	40
876	KLA	2	TC	PSME	90	1	40
877	KLA	2	TC	PSME	119	1	40
878	KLA	2	SC	PSME	94	1	40
879	KLA	2	PL	PSME	103	1	40
880	KLA	2	TC	PSME	154	1	41
881	KLA	2	PL	PSME	117	1	41
882	KLA	2	PL	PSME	127	1	41
883	KLA	2	PL	PSME	140	1	41
884	KLA	2	PL	PSME	107	1	42
885	KLA	2	PL	PSME	104	1	42
886	KLA	2	PL	PSME	94	1	42
887	KLA	2	PL	PSME	109	1	42
888	KLA	2	TC	PSME	102	1	43
889	KLA	2	TC	PSME	137	1	43
890	KLA	2	PL	PSME	76	1	43
891	KLA	2	SC	PSME	132	1	43
892	KLA	2	PL	PSME	102	1	43
893	KLA	2	TC	PSME	152	1	43
894	KLA	2	PL	PSME	117	1	43
895	KLA	2	TC	PSME	116	1	43
896	KLA	2	PL	PSME	93	1	44
897	KLA	2	TC	PSME	99	1	44
898	KLA	2	PL	PSME	130	1	44
899	KLA	2	PL	PSME	124	1	44
900	KLA	2	PL	PSME	84	1	44
901	KLA	2	PL	PSME	137	1	44
902	KLA	2	SC	PSME	54	1	44
903	KLA	2	TC	PSME	141	1	44
904	KLA	2	PL	PSME	117	1	45
905	KLA	2	PL	PSME	98	1	45
906	KLA	2	TC	PSME	130	1	46
907	KLA	2	PL	PSME	104	1	46
908	KLA	2	PL	PSME	117	1	46
909	KLA	2	PL	PIPO	103	1	46
910	KLA	2	PL	PSME	114	1	46
911	KLA	2	PL	PSME	135	1	46
912	KLA	2	PL	PSME	122	1	46
913	KLA	2	PL	PSME	152	1	46
914	KLA	2	TC	PSME	132	1	46
915	KLA	2	PL	PSME	155	1	46
916	KLA	2	PL	PSME	147	1	46
917	KLA	2	PL	PSME	127	1	46
918	KLA	2	PL	PSME	132	1	46
919	KLA	2	PL	PSME	109	1	46

920	KLA	2	PL	PSME	104	1	47
921	KLA	2	PL	PSME	104	1	47
922	KLA	2	PL	PSME	13	1	47
923	KLA	2	TC	PSME	109	1	47
924	KLA	2	SC	PSME	145	1	48
925	KLA	2	PL	PSME	100	1	49
926	KLA	2	TC	PSME	137	1	49
927	KLA	2	PL	PSME	114	1	49
928	KLA	2	PL	PSME	155	1	49
929	KLA	2	PL	PSME	150	1	49
930	KLA	2	SC	PSME	127	1	49
931	KLA	2	SC	PSME	117	1	49
932	KLA	2	PL	PSME	117	1	50
933	KLA	2	PL	PSME	124	1	50
934	KLA	2	PL	PSME	93	1	50
935	KLA	2	PL	CADE	173	1	51
936	KLA	2	PL	PSME	157	1	51
937	KLA	2	PL	PSME	165	1	52
938	KLA	2	PL	PSME	114	1	52
939	KLA	2	SC	PSME	141	1	52
940	KLA	2	PL	PSME	137	1	52
941	KLA	2	PL	PSME	122	1	53
942	KLA	2	PL	PSME	124	1	53
943	KLA	2	PL	PSME	142	1	53
944	KLA	2	PL	PSME	132	1	54
945	KLA	2	PL	PSME	170	1	55
946	KLA	2	PL	PSME	137	1	55
947	KLA	2	PL	PSME	127	1	55
948	KLA	2	PL	PSME	132	1	55
949	KLA	2	PL	PSME	122	1	55
950	KLA	2	PL	PSME	169	1	56
951	KLA	2	PL	PSME	97	1	56
952	KLA	2	TC	PSME	226	1	58
953	KLA	2	SC	PSME	145	1	60
954	KLA	2	PL	PSME	127	1	61
955	KLA	2	PL	PSME	144	1	61
956	KLA	2	PL	PSME	139	1	61
957	KLA	2	PL	PSME	126	1	62
958	KLA	2	PL	PSME	135	1	65
959	KLA	2	PL	PSME	140	1	65
960	KLA	2	TC	PSME	135	2	16
961	KLA	2	TC	PSME	69	2	19
962	KLA	2	TC	PSME	76	2	19
963	KLA	2	TC	PSME	74	2	20
964	KLA	2	SC	PSME	94	2	21
965	KLA	2	TC	PSME	180	2	21
966	KLA	2	TC	PSME	105	2	22
967	KLA	2	TC	PSME	15	2	23
968	KLA	2	TC	PSME	114	2	23
969	KLA	2	TC	PSME	84	2	24
970	KLA	2	TC	PSME	113	2	24
971	KLA	2	SC	PSME	98	2	24
972	KLA	2	TC	PSME	122	2	25
973	KLA	2	TC	PSME	124	2	25
974	KLA	2	TC	PSME	160	2	25
975	KLA	2	TC	PSME	120	2	26
976	KLA	2	TC	PSME	112	2	26
977	KLA	2	TC	PSME	94	2	27
978	KLA	2	TC	PSME	76	2	27
979	KLA	2	TC	PSME	93	2	28
980	KLA	2	TC	PSME	94	2	28
981	KLA	2	TC	PSME	91	2	29
982	KLA	2	SC	PSME	127	2	29
983	KLA	2	TC	PSME	100	2	29
984	KLA	2	TC	PSME	147	2	29
985	KLA	2	SC	PSME	147	2	29

986	KLA	2	TC	PSME	112	2	29
987	KLA	2	TC	PSME	117	2	30
988	KLA	2	PL	PSME	53	2	30
989	KLA	2	TC	PSME	109	2	30
990	KLA	2	TC	PSME	127	2	30
991	KLA	2	TC	PSME	100	2	30
992	KLA	2	TC	PSME	135	2	30
993	KLA	2	TC	PSME	89	2	31
994	KLA	2	TC	PSME	97	2	31
995	KLA	2	TC	PSME	119	2	31
996	KLA	2	TC	PSME	93	2	31
997	KLA	2	PL	PSME	110	2	31
998	KLA	2	TC	PSME	107	2	31
999	KLA	2	PL	PSME	142	2	31
1000	KLA	2	TC	PSME	117	2	32
1001	KLA	2	TC	ABGR	62	2	32
1002	KLA	2	TC	PSME	170	2	32
1003	KLA	2	TC	PSME	122	2	32
1004	KLA	2	TC	PSME	130	2	32
1005	KLA	2	TC	PSME	136	2	32
1006	KLA	2	TC	PSME	130	2	32
1007	KLA	2	PL	PSME	132	2	32
1008	KLA	2	TC	PSME	121	2	32
1009	KLA	2	TC	PSME	89	2	32
1010	KLA	2	TC	PSME	102	2	32
1011	KLA	2	TC	PSME	91	2	33
1012	KLA	2	TC	PSME	100	2	33
1013	KLA	2	TC	PSME	122	2	33
1014	KLA	2	TC	PSME	79	2	33
1015	KLA	2	TC	PSME	124	2	33
1016	KLA	2	TC	PSME	142	2	34
1017	KLA	2	TC	PSME	152	2	34
1018	KLA	2	TC	PSME	76	2	34
1019	KLA	2	TC	PSME	71	2	34
1020	KLA	2	TC	PSME	130	2	34
1021	KLA	2	TC	PSME	104	2	34
1022	KLA	2	TC	PSME	124	2	34
1023	KLA	2	TC	PSME	137	2	34
1024	KLA	2	TC	PSME	102	2	34
1025	KLA	2	SC	PSME	104	2	34
1026	KLA	2	TC	PSME	140	2	34
1027	KLA	2	TC	PSME	117	2	35
1028	KLA	2	TC	PSME	114	2	35
1029	KLA	2	TC	PSME	139	2	35
1030	KLA	2	TC	PSME	114	2	35
1031	KLA	2	TC	PSME	117	2	35
1032	KLA	2	TC	PSME	132	2	35
1033	KLA	2	TC	PSME	102	2	35
1034	KLA	2	TC	PSME	130	2	36
1035	KLA	2	TC	PSME	119	2	36
1036	KLA	2	TC	PSME	122	2	36
1037	KLA	2	TC	PSME	124	2	36
1038	KLA	2	PL	PSME	94	2	36
1039	KLA	2	TC	PSME	98	2	36
1040	KLA	2	TC	PSME	117	2	36
1041	KLA	2	TC	PSME	117	2	36
1042	KLA	2	TC	PSME	109	2	37
1043	KLA	2	TC	PSME	150	2	37
1044	KLA	2	TC	PSME	179	2	37
1045	KLA	2	TC	PSME	173	2	37
1046	KLA	2	PL	PSME	179	2	37
1047	KLA	2	TC	PSME	131	2	37
1048	KLA	2	TC	PIJE	104	2	37
1049	KLA	2	TC	PSME	100	2	38
1050	KLA	2	TC	PSME	129	2	38
1051	KLA	2	TC	PSME	158	2	38

1052	KLA	2	TC	PSME	119	2	38
1053	KLA	2	TC	PSME	84	2	38
1054	KLA	2	TC	PSME	117	2	38
1055	KLA	2	SC	PSME	174	2	38
1056	KLA	2	TC	PSME	165	2	38
1057	KLA	2	TC	PSME	124	2	39
1058	KLA	2	TC	PSME	117	2	39
1059	KLA	2	TC	PSME	160	2	39
1060	KLA	2	TC	PSME	109	2	39
1061	KLA	2	TC	PSME	136	2	39
1062	KLA	2	TC	PSME	137	2	39
1063	KLA	2	TC	PSME	145	2	39
1064	KLA	2	TC	PSME	94	2	39
1065	KLA	2	TC	PSME	165	2	39
1066	KLA	2	TC	PSME	112	2	40
1067	KLA	2	SC	PSME	115	2	40
1068	KLA	2	TC	PSME	144	2	40
1069	KLA	2	TC	PSME	109	2	40
1070	KLA	2	TC	PSME	119	2	40
1071	KLA	2	PL	PSME	123	2	40
1072	KLA	2	TC	PSME	170	2	41
1073	KLA	2	TC	PSME	132	2	41
1074	KLA	2	TC	PSME	150	2	41
1075	KLA	2	TC	PSME	119	2	41
1076	KLA	2	TC	PSME	155	2	41
1077	KLA	2	TC	PSME	170	2	41
1078	KLA	2	TC	PSME	79	2	41
1079	KLA	2	TC	PSME	114	2	41
1080	KLA	2	TC	PSME	142	2	41
1081	KLA	2	TC	PSME	145	2	41
1082	KLA	2	TC	PSME	135	2	42
1083	KLA	2	PL	PSME	133	2	42
1084	KLA	2	TC	PSME	135	2	43
1085	KLA	2	TC	PSME	113	2	43
1086	KLA	2	TC	PSME	150	2	43
1087	KLA	2	TC	PSME	169	2	43
1088	KLA	2	TC	PSME	135	2	43
1089	KLA	2	TC	PSME	64	2	43
1090	KLA	2	TC	PSME	124	2	44
1091	KLA	2	TC	PSME	94	2	44
1092	KLA	2	TC	PSME	130	2	44
1093	KLA	2	TC	PSME	118	2	44
1094	KLA	2	TC	PSME	152	2	44
1095	KLA	2	TC	PSME	160	2	45
1096	KLA	2	TC	PSME	170	2	45
1097	KLA	2	TC	PSME	117	2	45
1098	KLA	2	TC	PSME	154	2	45
1099	KLA	2	TC	PSME	147	2	45
1100	KLA	2	TC	PSME	147	2	46
1101	KLA	2	TC	PSME	146	2	47
1102	KLA	2	TC	PSME	112	2	47
1103	KLA	2	TC	PSME	153	2	48
1104	KLA	2	TC	PSME	130	2	48
1105	KLA	2	TC	PSME	141	2	49
1106	KLA	2	TC	PSME	114	2	49
1107	KLA	2	TC	PSME	24	2	49
1108	KLA	2	SC	PSME	104	2	49
1109	KLA	2	TC	PSME	173	2	49
1110	KLA	2	TC	PSME	132	2	49
1111	KLA	2	TC	PSME	132	2	49
1112	KLA	2	TC	PSME	116	2	50
1113	KLA	2	TC	PSME	140	2	51
1114	KLA	2	TC	PSME	144	2	51
1115	KLA	2	TC	PSME	132	2	52
1116	KLA	2	TC	PSME	163	2	52
1117	KLA	2	TC	PSME	152	2	52

1118	KLA	2	SC	PSME	144	2	52
1119	KLA	2	TC	PSME	150	2	54
1120	KLA	2	SC	PSME	131	2	55
1121	KLA	2	PL	PSME	147	2	55
1122	KLA	2	TC	PSME	151	2	58
1123	KLA	2	TC	PSME	162	2	58
1124	KLA	2	TC	PSME	178	2	60
1125	KLA	2	PL	PSME	200	2	63
1126	KLA	2	TC	PSME	177	2	71
1127	KLA	2	SC	PSME	131	3	13
1128	KLA	2	TC	PILA	55	3	14
1129	KLA	2	TC	PSME	132	3	14
1130	KLA	2	SC	PSME	32	3	15
1131	KLA	2	TC	PSME	113	3	17
1132	KLA	2	TC	PSME	160	3	17
1133	KLA	2	TC	PSME	127	3	18
1134	KLA	2	SC	PSME	91	3	18
1135	KLA	2	TC	PSME	109	3	20
1136	KLA	2	SC	PSME	132	3	23
1137	KLA	2	SC	PSME	134	3	30
1138	KLA	2	SC	PSME	140	3	30
1139	KLA	2	SC	PSME	147	3	41
1140	KLA	2	TC	PSME	132	4	7
1141	KLA	2	SC	PSME	147	4	14
1142	KLA	2	TC	PSME	93	4	18
1143	KLA	2	TC	PSME	130	4	21
1144	KLA	2	TC	PSME	137	4	24
1145	KLA	2	SC	PSME	150	4	26
1146	KLA	2	SC	PSME	112	4	32
1147	KLA	2	SC	PSME	123	4	44
1148	KLA	2	SC	PSME	61	6	9
1149	KLA	2	TC	PSME	99	6	15
1150	KLA	2	SC	PSME	84	6	16
1151	KLA	2	TC	PSME	122	6	22
1152	KLA	2	TC	PSME	114	7	14
1153	TYE	2	TC	PSME	122	2	36
1154	TYE	2	TC	PSME	107	2	35
1155	TYE	2	TC	PSME	109	2	35
1156	TYE	2	TC	PSME	112	2	35
1157	TYE	2	TC	PSME	117	2	35
1158	TYE	2	TC	PSME	117	2	35
1159	TYE	2	TC	PSME	132	2	35
1160	TYE	2	TC	PSME	137	2	35
1161	TYE	2	TC	PSME	141	2	35
1162	TYE	2	TC	PSME	159	2	35
1163	TYE	2	TC	PSME	168	2	35
1164	TYE	2	TC	PSME	175	2	35
1165	TYE	2	TC	PSME	95	2	36
1166	TYE	2	TC	PSME	155	2	37
1167	TYE	2	TC	PSME	109	2	36
1168	TYE	2	TC	PSME	152	2	34
1169	TYE	2	TC	PSME	137	2	36
1170	TYE	2	TC	PSME	138	2	36
1171	TYE	2	TC	PSME	145	2	36
1172	TYE	2	TC	PSME	145	2	36
1173	TYE	2	TC	PSME	152	2	36
1174	TYE	2	TC	PSME	159	6	36
1175	TYE	2	TC	PSME	165	2	36
1176	TYE	2	TC	PSME	89	2	37
1177	TYE	2	TC	PSME	91	2	37
1178	TYE	2	TC	PSME	94	2	37
1179	TYE	2	TC	PSME	101	2	37
1180	TYE	2	TC	PSME	113	2	37
1181	TYE	2	TC	PSME	127	2	32
1182	TYE	2	TC	TSHE	95	2	36
1183	TYE	2	TC	PSME	162	2	33

1184	TYE	2	TC	PSME	127	2	42
1185	TYE	2	TC	PSME	148	2	32
1186	TYE	2	TC	PSME	175	2	32
1187	TYE	2	TC	PSME	71	2	33
1188	TYE	2	TC	PSME	89	2	33
1189	TYE	2	TC	PSME	102	2	33
1190	TYE	2	TC	PSME	109	2	33
1191	TYE	2	TC	PSME	117	2	33
1192	TYE	2	TC	PSME	118	2	33
1193	TYE	2	TC	PSME	132	2	33
1194	TYE	2	TC	PSME	143	2	33
1195	TYE	2	TC	PSME	145	2	33
1196	TYE	2	TC	PSME	145	2	33
1197	TYE	2	TC	PSME	107	2	35
1198	TYE	2	TC	PSME	142	2	34
1199	TYE	2	TC	PSME	161	2	37
1200	TYE	2	TC	PSME	152	2	34
1201	TYE	2	TC	PSME	150	2	34
1202	TYE	2	TC	PSME	143	2	34
1203	TYE	2	TC	PSME	142	2	34
1204	TYE	2	TC	PSME	147	2	33
1205	TYE	2	TC	PSME	142	2	34
1206	TYE	2	TC	PSME	155	2	33
1207	TYE	2	TC	PSME	130	2	34
1208	TYE	2	TC	PSME	126	2	34
1209	TYE	2	TC	PSME	122	2	34
1210	TYE	2	TC	PSME	106	2	34
1211	TYE	2	TC	PSME	180	2	33
1212	TYE	2	TC	PSME	157	2	34
1213	TYE	2	TC	PSME	142	2	34
1214	TYE	2	TC	PSME	127	2	41
1215	TYE	2	TC	PSME	62	2	6
1216	TYE	2	TC	PSME	122	2	40
1217	TYE	2	TC	PSME	142	2	40
1218	TYE	2	TC	PSME	146	2	40
1219	TYE	2	TC	PSME	152	2	40
1220	TYE	2	TC	PSME	152	2	40
1221	TYE	2	TC	PSME	153	2	40
1222	TYE	2	TC	PSME	163	2	40
1223	TYE	2	TC	PSME	173	2	40
1224	TYE	2	TC	PSME	188	2	40
1225	TYE	2	TC	PSME	76	2	41
1226	TYE	2	TC	PSME	109	2	41
1227	TYE	2	TC	PSME	117	2	37
1228	TYE	2	TC	PSME	122	2	41
1229	TYE	2	TC	PSME	216	2	39
1230	TYE	2	TC	PSME	132	2	41
1231	TYE	2	TC	PSME	133	2	41
1232	TYE	2	TC	PSME	141	2	41
1233	TYE	2	TC	PSME	150	2	41
1234	TYE	2	TC	PSME	152	2	41
1235	TYE	2	TC	PSME	175	2	41
1236	TYE	2	TC	PSME	180	2	41
1237	TYE	2	TC	PSME	99	2	42
1238	TYE	2	TC	PSME	109	2	42
1239	TYE	2	TC	PSME	110	2	42
1240	TYE	2	TC	PSME	116	2	42
1241	TYE	2	TC	PSME	117	2	42
1242	TYE	2	TC	PSME	121	2	42
1243	TYE	2	TC	PSME	114	2	41
1244	TYE	2	TC	PSME	144	2	38
1245	TYE	2	TC	PSME	172	2	37
1246	TYE	2	TC	PSME	180	2	37
1247	TYE	2	TC	PSME	183	2	37
1248	TYE	2	TC	PSME	88	2	38
1249	TYE	2	TC	PSME	102	2	38

1250	TYE	2	TC	PSME	103	2	38
1251	TYE	2	TC	PSME	122	2	38
1252	TYE	2	TC	PSME	127	2	38
1253	TYE	2	TC	PSME	127	2	38
1254	TYE	2	TC	PSME	130	2	38
1255	TYE	2	TC	PSME	135	2	38
1256	TYE	2	TC	PSME	139	2	38
1257	TYE	2	TC	PSME	141	2	38
1258	TYE	2	TC	PSME	113	2	40
1259	TYE	2	TC	PSME	123	2	39
1260	TYE	2	TC	PSME	122	2	32
1261	TYE	2	TC	PSME	203	2	39
1262	TYE	2	TC	PSME	160	2	39
1263	TYE	2	TC	PSME	142	2	39
1264	TYE	2	TC	PSME	141	2	39
1265	TYE	2	TC	PSME	141	2	38
1266	TYE	2	TC	THPL	124	2	39
1267	TYE	2	TC	PSME	141	2	38
1268	TYE	2	TC	PSME	114	2	39
1269	TYE	2	TC	PSME	104	2	39
1270	TYE	2	TC	PSME	219	2	38
1271	TYE	2	TC	PSME	165	2	38
1272	TYE	2	TC	PSME	149	1	38
1273	TYE	2	TC	PSME	96	2	40
1274	TYE	2	TC	PSME	137	2	39
1275	TYE	2	TC	PSME	141	2	21
1276	TYE	2	TC	PSME	70	6	18
1277	TYE	2	TC	PSME	99	4	18
1278	TYE	2	TC	PSME	102	7	18
1279	TYE	2	TC	PSME	137	7	18
1280	TYE	2	TC	PSME	142	6	18
1281	TYE	2	TC	PSME	155	2	18
1282	TYE	2	TC	PSME	79	2	19
1283	TYE	2	TC	PSME	105	2	19
1284	TYE	2	TC	PSME	132	2	19
1285	TYE	2	TC	PSME	85	2	20
1286	TYE	2	TC	PSME	97	3	20
1287	TYE	2	TC	PSME	97	2	20
1288	TYE	2	TC	PSME	201	6	23
1289	TYE	2	TC	PSME	109	2	21
1290	TYE	2	TC	PSME	107	2	17
1291	TYE	2	TC	PSME	82	2	22
1292	TYE	2	TC	PSME	97	2	22
1293	TYE	2	TC	PSME	102	2	22
1294	TYE	2	TC	PSME	106	2	22
1295	TYE	2	TC	THPL	114	2	22
1296	TYE	2	TC	PSME	125	2	22
1297	TYE	2	TC	PSME	137	2	22
1298	TYE	2	TC	THPL	89	2	23
1299	TYE	2	TC	PSME	91	2	23
1300	TYE	2	TC	PSME	94	2	23
1301	TYE	2	TC	PSME	99	2	23
1302	TYE	2	TC	PSME	108	5	23
1303	TYE	2	TC	PSME	136	2	32
1304	TYE	2	TC	PSME	99	2	20
1305	TYE	2	TC	PSME	180	6	13
1306	TYE	2	TC	PSME	81	6	7
1307	TYE	2	TC	PSME	91	7	10
1308	TYE	2	TC	PSME	71	6	11
1309	TYE	2	TC	PSME	98	2	11
1310	TYE	2	TC	PSME	102	6	11
1311	TYE	2	TC	PSME	76	4	12
1312	TYE	2	TC	PSME	87	7	12
1313	TYE	2	TC	PSME	113	5	12
1314	TYE	2	TC	PSME	127	7	12
1315	TYE	2	TC	PSME	142	3	12

1316	TYE	2	TC	PSME	94	7	13
1317	TYE	2	TC	PSME	105	4	13
1318	TYE	2	TC	PSME	106	3	13
1319	TYE	2	TC	THPL	118	6	17
1320	TYE	2	TC	PSME	69	3	16
1321	TYE	2	TC	PSME	91	2	24
1322	TYE	2	TC	PSME	101	7	17
1323	TYE	2	TC	PSME	91	6	17
1324	TYE	2	TC	PSME	57	2	17
1325	TYE	2	TC	PSME	124	6	16
1326	TYE	2	TC	PSME	125	6	13
1327	TYE	2	TC	PSME	86	2	16
1328	TYE	2	TC	PSME	163	3	13
1329	TYE	2	TC	PSME	185	7	15
1330	TYE	2	TC	PSME	94	7	15
1331	TYE	2	TC	PSME	108	6	14
1332	TYE	2	TC	PSME	107	6	14
1333	TYE	2	TC	PSME	102	6	14
1334	TYE	2	TC	PSME	110	4	17
1335	TYE	2	TC	PSME	116	4	16
1336	TYE	2	TC	PSME	132	6	30
1337	TYE	2	TC	PSME	146	2	28
1338	TYE	2	TC	PSME	94	2	29
1339	TYE	2	TC	PSME	94	2	29
1340	TYE	2	TC	PSME	114	2	29
1341	TYE	2	TC	PSME	122	2	29
1342	TYE	2	TC	PSME	127	2	29
1343	TYE	2	TC	PSME	130	2	29
1344	TYE	2	TC	PSME	159	2	29
1345	TYE	2	TC	PSME	196	2	29
1346	TYE	2	TC	PSME	104	2	30
1347	TYE	2	TC	THPL	111	2	30
1348	TYE	2	TC	PSME	121	2	30
1349	TYE	2	TC	PSME	140	2	23
1350	TYE	2	TC	PSME	130	2	30
1351	TYE	2	TC	PSME	107	2	28
1352	TYE	2	TC	PSME	145	2	30
1353	TYE	2	TC	PSME	180	2	30
1354	TYE	2	TC	PSME	197	2	30
1355	TYE	2	TC	PSME	83	2	31
1356	TYE	2	TC	PSME	110	2	31
1357	TYE	2	TC	PSME	114	2	31
1358	TYE	2	TC	PSME	120	2	31
1359	TYE	2	TC	PSME	122	2	31
1360	TYE	2	TC	PSME	124	2	31
1361	TYE	2	TC	PSME	132	2	31
1362	TYE	2	TC	PSME	193	2	31
1363	TYE	2	TC	PSME	90	2	32
1364	TYE	2	TC	PSME	106	2	32
1365	TYE	2	TC	PSME	127	2	30
1366	TYE	2	TC	PSME	109	2	26
1367	TYE	2	TC	PSME	94	6	24
1368	TYE	2	TC	PSME	94	2	24
1369	TYE	2	TC	PSME	104	2	24
1370	TYE	2	TC	PSME	109	2	24
1371	TYE	2	TC	PSME	110	6	24
1372	TYE	2	TC	PSME	112	4	24
1373	TYE	2	TC	PSME	84	2	25
1374	TYE	2	TC	PSME	99	2	25
1375	TYE	2	TC	PSME	114	2	25
1376	TYE	2	TC	PSME	134	2	25
1377	TYE	2	TC	PSME	141	2	25
1378	TYE	2	TC	PSME	148	2	25
1379	TYE	2	TC	PSME	150	2	25
1380	TYE	2	TC	PSME	142	2	28
1381	TYE	2	TC	PSME	100	2	27

1382	TYE	2	TC	PSME	127	2	40
1383	TYE	2	TC	PSME	95	2	28
1384	TYE	2	TC	PSME	138	2	27
1385	TYE	2	TC	PSME	119	2	27
1386	TYE	2	TC	PSME	115	2	27
1387	TYE	2	TC	PSME	98	6	26
1388	TYE	2	TC	PSME	108	2	27
1389	TYE	2	TC	PSME	108	2	26
1390	TYE	2	TC	PSME	81	2	27
1391	TYE	2	TC	PSME	59	6	27
1392	TYE	2	TC	PSME	124	2	26
1393	TYE	2	TC	PSME	115	2	26
1394	TYE	2	TC	PSME	110	2	26
1395	TYE	2	TC	PSME	127	2	28
1396	TYE	2	TC	PSME	114	2	27
1397	TYE	2	TC	PSME	84	2	51
1398	TYE	2	TC	PSME	177	2	49
1399	TYE	2	TC	PSME	180	2	49
1400	TYE	2	TC	PSME	182	2	49
1401	TYE	2	TC	PSME	125	1	50
1402	TYE	2	TC	PSME	135	2	50
1403	TYE	2	TC	PSME	140	2	50
1404	TYE	2	TC	PSME	146	2	50
1405	TYE	2	TC	PSME	150	2	50
1406	TYE	2	TC	PSME	157	2	50
1407	TYE	2	TC	PSME	161	2	50
1408	TYE	2	TC	PSME	170	2	50
1409	TYE	2	TC	PSME	183	2	47
1410	TYE	2	TC	PSME	172	2	50
1411	TYE	2	TC	PSME	156	2	49
1412	TYE	2	TC	PSME	107	1	51
1413	TYE	2	TC	PSME	150	2	51
1414	TYE	2	TC	PSME	153	2	51
1415	TYE	2	TC	PSME	161	2	51
1416	TYE	2	TC	PSME	161	2	51
1417	TYE	2	TC	PSME	170	2	51
1418	TYE	2	TC	PSME	180	2	51
1419	TYE	2	TC	PSME	132	2	52
1420	TYE	2	TC	PSME	152	2	52
1421	TYE	2	TC	PSME	171	2	52
1422	TYE	2	TC	PSME	200	2	52
1423	TYE	2	TC	PSME	213	2	52
1424	TYE	2	TC	PSME	171	2	50
1425	TYE	2	TC	PSME	185	2	48
1426	TYE	2	TC	PSME	189	2	47
1427	TYE	2	TC	PSME	193	2	47
1428	TYE	2	TC	PSME	129	2	48
1429	TYE	2	TC	PSME	130	2	48
1430	TYE	2	TC	PSME	132	2	48
1431	TYE	2	TC	PSME	142	2	48
1432	TYE	2	TC	PSME	144	2	48
1433	TYE	2	TC	PSME	149	2	48
1434	TYE	2	TC	PSME	152	2	48
1435	TYE	2	TC	PSME	159	2	48
1436	TYE	2	TC	PSME	168	2	48
1437	TYE	2	TC	PSME	170	2	48
1438	TYE	2	TC	PSME	159	2	49
1439	TYE	2	TC	PSME	181	2	48
1440	TYE	2	TC	PSME	122	2	40
1441	TYE	2	TC	PSME	190	2	48
1442	TYE	2	TC	PSME	190	2	48
1443	TYE	2	TC	PSME	89	2	49
1444	TYE	2	TC	PSME	104	2	49
1445	TYE	2	TC	PSME	108	2	49
1446	TYE	2	TC	PSME	135	2	49
1447	TYE	2	TC	PSME	137	2	49

1448	TYE	2	TC	PSME	142	2	49
1449	TYE	2	TC	PSME	150	2	49
1450	TYE	2	TC	PSME	152	2	49
1451	TYE	2	TC	PSME	133	2	42
1452	TYE	2	TC	PSME	142	2	53
1453	TYE	2	TC	PSME	178	2	48
1454	TYE	2	TC	PSME	196	2	61
1455	TYE	2	TC	PSME	122	2	53
1456	TYE	2	TC	PSME	165	2	57
1457	TYE	2	TC	PSME	190	2	57
1458	TYE	2	TC	PSME	229	2	57
1459	TYE	2	TC	PSME	147	2	58
1460	TYE	2	TC	PSME	172	2	58
1461	TYE	2	TC	PSME	185	2	59
1462	TYE	2	TC	PSME	202	2	59
1463	TYE	2	TC	PSME	127	2	60
1464	TYE	2	TC	PSME	183	2	60
1465	TYE	2	TC	PSME	191	2	60
1466	TYE	2	TC	PSME	152	2	61
1467	TYE	2	TC	PSME	130	2	57
1468	TYE	2	TC	PSME	175	2	61
1469	TYE	2	TC	PSME	190	2	56
1470	TYE	2	TC	PSME	203	2	61
1471	TYE	2	TC	PSME	140	2	62
1472	TYE	2	TC	PSME	224	2	62
1473	TYE	2	TC	PSME	122	2	63
1474	TYE	2	TC	PSME	243	2	64
1475	TYE	2	TC	PSME	147	2	65
1476	TYE	2	TC	PSME	188	2	65
1477	TYE	2	TC	PSME	170	2	66
1478	TYE	2	TC	PSME	209	2	66
1479	TYE	2	TC	PSME	160	2	67
1480	TYE	2	TC	PSME	246	2	67
1481	TYE	2	TC	PSME	177	2	69
1482	TYE	2	TC	PSME	160	2	61
1483	TYE	2	TC	PSME	168	2	54
1484	TYE	2	TC	PSME	152	2	49
1485	TYE	2	TC	PSME	147	2	53
1486	TYE	2	TC	PSME	149	2	53
1487	TYE	2	TC	PSME	161	2	53
1488	TYE	2	TC	PSME	168	2	53
1489	TYE	2	TC	PSME	173	2	53
1490	TYE	2	TC	PSME	180	2	53
1491	TYE	2	TC	PSME	197	2	53
1492	TYE	2	TC	PSME	199	2	53
1493	TYE	2	TC	PSME	91	2	54
1494	TYE	2	TC	PSME	130	2	54
1495	TYE	2	TC	PSME	134	2	54
1496	TYE	2	TC	PSME	155	2	57
1497	TYE	2	TC	PSME	158	2	54
1498	TYE	2	TC	PSME	138	2	53
1499	TYE	2	TC	PSME	170	2	54
1500	TYE	2	TC	PSME	135	2	55
1501	TYE	2	TC	PSME	146	2	55
1502	TYE	2	TC	PSME	147	2	55
1503	TYE	2	TC	PSME	163	2	55
1504	TYE	2	TC	PSME	164	2	55
1505	TYE	2	TC	PSME	178	2	55
1506	TYE	2	TC	PSME	193	2	55
1507	TYE	2	TC	PSME	196	2	55
1508	TYE	2	TC	PSME	114	2	56
1509	TYE	2	TC	PSME	143	2	56
1510	TYE	2	TC	PSME	171	2	56
1511	TYE	2	TC	PSME	152	2	54
1512	TYE	2	TC	PSME	159	2	43
1513	TYE	2	TC	PSME	155	2	46

1514	TYE	2	TC	PSME	160	2	46
1515	TYE	2	TC	PSME	175	2	46
1516	TYE	2	TC	PSME	185	2	46
1517	TYE	2	TC	PSME	88	2	44
1518	TYE	2	TC	PSME	120	2	47
1519	TYE	2	TC	PSME	160	2	43
1520	TYE	2	TC	PSME	141	2	45
1521	TYE	2	TC	THPL	157	2	43
1522	TYE	2	TC	PSME	135	2	44
1523	TYE	2	TC	PSME	142	2	43
1524	TYE	2	TC	PSME	148	2	46
1525	TYE	2	TC	PSME	137	2	45
1526	TYE	2	TC	PSME	142	2	43
1527	TYE	2	TC	PSME	153	2	44
1528	TYE	2	TC	PSME	135	2	46
1529	TYE	2	TC	PSME	135	2	44
1530	TYE	2	TC	THPL	244	2	45
1531	TYE	2	TC	PSME	106	2	46
1532	TYE	2	TC	PSME	127	2	44
1533	TYE	2	TC	PSME	117	2	46
1534	TYE	2	TC	PSME	137	2	44
1535	TYE	2	TC	PSME	152	2	46
1536	TYE	2	TC	PSME	180	2	45
1537	TYE	2	TC	PSME	140	2	45
1538	TYE	2	TC	PSME	164	2	45
1539	TYE	2	TC	PSME	137	2	44
1540	TYE	2	TC	PSME	144	2	44
1541	TYE	2	TC	PSME	113	2	44
1542	TYE	2	TC	PSME	147	2	44
1543	TYE	2	TC	PSME	118	2	47
1544	TYE	2	TC	PSME	134	2	46
1545	TYE	2	TC	PSME	135	2	42
1546	TYE	2	TC	PSME	109	2	45
1547	TYE	2	TC	PSME	120	2	47
1548	TYE	2	TC	PSME	154	2	47
1549	TYE	2	TC	PSME	114	2	47
1550	TYE	2	TC	PSME	163	2	42
1551	TYE	2	TC	PSME	157	2	42
1552	TYE	2	TC	PSME	154	2	47
1553	TYE	2	TC	PSME	188	2	44
1554	TYE	2	TC	PSME	173	2	47
1555	TYE	2	TC	PSME	106	2	45
1556	TYE	2	TC	PSME	52	2	45
1557	TYE	2	TC	PSME	160	2	47
1558	TYE	2	TC	PSME	160	2	47
1559	TYE	2	TC	PSME	170	2	47
1560	TYE	2	TC	PSME	170	2	47
1561	TYE	2	TC	PSME	141	2	42
1562	TYE	2	TC	PSME	164	2	42
1563	TYE	2	TC	PSME	159	2	44
1564	TYE	2	TC	PSME	160	2	44
1565	TYE	2	TC	PSME	167	2	42
1566	TYE	2	TC	PSME	132	2	47
1567	TYE	2	TC	PSME	182	2	44
1568	TYE	2	TC	PSME	133	2	47
1569	TYE	2	TC	PSME	153	2	47
1570	TYE	2	TC	PSME	150	2	47
1571	TYE	2	TC	PSME	187	2	44
1572	TYE	2	TC	THPL	150	2	47
1573	TYE	2	TC	PSME	164	2	42
1574	TYE	2	SC	PSME	198	2	54
1575	TYE	2	SC	ARME	72	1	23
1576	TYE	2	SC	PSME	75	6	23
1577	TYE	2	SC	PSME	201	2	53
1578	TYE	2	SC	PSME	61	2	25
1579	TYE	2	SC	PSME	130	2	40

1580	TYE	2	SC	PSME	103	2	40
1581	TYE	2	SC	PSME	274	2	54
1582	TYE	2	SC	PSME	107	4	24
1583	TYE	2	SC	PSME	165	7	23
1584	TYE	2	SC	PSME	154	1	55
1585	TYE	2	SC	PSME	107	1	54
1586	TYE	2	SC	PSME	79	2	25
1587	TYE	2	SC	PSME	167	7	13
1588	TYE	2	SC	ARME	67	2	20
1589	TYE	2	SC	PSME	139	2	61
1590	TYE	2	SC	THPL	89	6	17
1591	TYE	2	SC	THPL	138	2	43
1592	TYE	2	SC	PSME	124	1	43
1593	TYE	2	SC	PSME	156	1	62
1594	TYE	2	SC	THPL	147	6	18
1595	TYE	2	SC	PSME	188	2	63
1596	TYE	2	SC	PSME	188	2	40
1597	TYE	2	SC	PSME	239	2	63
1598	TYE	2	SC	PSME	144	2	42
1599	TYE	2	SC	PSME	124	2	42
1600	TYE	2	SC	PSME	127	2	42
1601	TYE	2	SC	PSME	137	2	70
1602	TYE	2	SC	PSME	95	7	9
1603	TYE	2	SC	PSME	193	1	62
1604	TYE	2	SC	THPL	168	1	40
1605	TYE	2	SC	THPL	130	2	44
1606	TYE	2	SC	PSME	152	2	40
1607	TYE	2	SC	PSME	130	7	21
1608	TYE	2	SC	CADE	197	2	56
1609	TYE	2	SC	ARME	82	1	21
1610	TYE	2	SC	PSME	109	7	20
1611	TYE	2	SC	PSME	238	2	43
1612	TYE	2	SC	PSME	121	2	44
1613	TYE	2	SC	PSME	145	2	40
1614	TYE	2	SC	PSME	160	1	57
1615	TYE	2	SC	PSME	252	1	44
1616	TYE	2	SC	PSME	112	2	44
1617	TYE	2	SC	PSME	108	1	39
1618	TYE	2	SC	PSME	106	4	19
1619	TYE	2	SC	PSME	105	7	19
1620	TYE	2	SC	PSME	107	2	20
1621	TYE	2	SC	PSME	142	2	46
1622	TYE	2	SC	PSME	138	3	38
1623	TYE	2	SC	PSME	156	2	47
1624	TYE	2	SC	PSME	63	1	50
1625	TYE	2	SC	PSME	188	2	34
1626	TYE	2	SC	PSME	166	2	36
1627	TYE	2	SC	THPL	101	1	30
1628	TYE	2	SC	TSHE	98	2	51
1629	TYE	2	SC	PSME	182	2	50
1630	TYE	2	SC	PSME	143	2	30
1631	TYE	2	SC	PSME	163	2	30
1632	TYE	2	SC	PSME	107	2	38
1633	TYE	2	SC	PSME	50	2	31
1634	TYE	2	SC	PSME	183	2	29
1635	TYE	2	SC	PSME	140	2	46
1636	TYE	2	SC	PSME	140	2	48
1637	TYE	2	SC	PSME	155	2	47
1638	TYE	2	SC	PSME	123	2	36
1639	TYE	2	SC	PSME	152	2	48
1640	TYE	2	SC	THPL	158	2	37
1641	TYE	2	SC	PSME	141	6	34
1642	TYE	2	SC	PSME	131	2	34
1643	TYE	2	SC	PSME	118	2	37
1644	TYE	2	SC	PSME	151	2	49
1645	TYE	2	SC	PSME	142	2	49

1646	TYE	2	SC	PSME	123	2	33
1647	TYE	2	SC	PSME	121	2	49
1648	TYE	2	SC	CADE	228	3	48
1649	TYE	2	SC	PSME	170	2	34
1650	TYE	2	SC	PSME	130	6	27
1651	TYE	2	SC	PSME	130	1	39
1652	TYE	2	SC	CADE	157	1	49
1653	TYE	2	SC	PSME	123	2	26
1654	TYE	2	SC	PSME	74	2	27
1655	TYE	2	SC	TSHE	83	2	39
1656	TYE	2	SC	PSME	194	2	47
1657	TYE	2	SC	PSME	145	2	53
1658	TYE	2	SC	PSME	136	2	45
1659	TYE	2	SC	THPL	94	1	53
1660	TYE	2	SC	PSME	77	1	28
1661	TYE	2	SC	PSME	154	2	38
1662	TYE	2	SC	PSME	130	1	52
1663	TYE	2	SC	PSME	147	2	29
1664	TYE	2	SC	THPL	140	2	38
1665	TYE	2	SC	PSME	201	2	51
1666	TYE	2	SC	PSME	147	2	38
1667	TYE	2	SC	PSME	147	2	45
1668	TYE	2	SC	ACMA	85	1	29
1669	TYE	2	SC	PSME	145	2	52
1670	TYE	2	SC	PSME	107	2	35
1671	TYE	2	PL	PSME	180	2	48
1672	TYE	2	PL	PSME	167	1	42
1673	TYE	2	PL	PSME	130	1	66
1674	TYE	2	PL	PSME	127	1	62
1675	TYE	2	PL	PSME	133	1	75
1676	TYE	2	PL	PSME	193	1	41
1677	TYE	2	PL	PSME	152	1	71
1678	TYE	2	PL	PSME	43	2	12
1679	TYE	2	PL	PSME	127	1	47
1680	TYE	2	PL	PSME	131	1	42
1681	TYE	2	PL	PSME	31	1	11
1682	TYE	2	PL	PSME	152	1	70
1683	TYE	2	PL	PSME	159	1	34
1684	TYE	2	PL	PSME	166	1	48
1685	TYE	2	PL	PSME	180	1	74
1686	TYE	2	PL	PSME	93	1	35
1687	TYE	2	PL	PSME	104	1	15
1688	TYE	2	PL	PSME	104	1	38
1689	TYE	2	PL	PSME	65	1	54
1690	TYE	2	PL	PSME	141	1	54
1691	TYE	2	PL	PSME	79	1	39
1692	TYE	2	PL	PSME	122	1	53
1693	TYE	2	PL	PSME	173	1	54
1694	TYE	2	PL	CADE	51	1	24
1695	TYE	2	PL	PSME	178	1	79
1696	TYE	2	PL	PSME	124	2	29
1697	TYE	2	PL	PSME	183	2	45
1698	TYE	2	PL	PSME	29	2	56
1699	TYE	2	PL	PSME	69	2	30
1700	TYE	2	PL	CADE	100	2	30
1701	TYE	2	PL	PSME	83	1	38
1702	TYE	2	PL	PSME	121	1	38
1703	TYE	2	PL	PSME	98	2	34
1704	TYE	2	PL	PSME	140	1	50
1705	TYE	2	PL	PSME	101	2	26
1706	TYE	2	PL	PSME	97	1	38
1707	TYE	2	PL	PSME	109	1	45
1708	TYE	2	PL	PSME	107	1	58
1709	TYE	2	PL	PSME	137	1	58
1710	TYE	2	PL	PSME	138	1	37
1711	TYE	2	PL	PSME	216	1	43

1712	TYE	2	PL	PSME	107	1	47
1713	TYE	2	PL	PSME	111	1	47
1714	TYE	2	PL	PSME	117	1	61
1715	TYE	2	PL	ABGR	93	1	37
1716	TYE	2	PL	PSME	84	2	37
1717	TYE	2	PL	PSME	158	1	56
